# Blood Group Antigen Recognition via the Group A Streptococcal M Protein Mediates Host Colonization

**DOI:** 10.1128/mBio.02237-16

**Published:** 2017-01-24

**Authors:** David M. P. De Oliveira, Lauren Hartley-Tassell, Arun Everest-Dass, Christopher J. Day, Rebecca A. Dabbs, Thomas Ve, Bostjan Kobe, Victor Nizet, Nicolle H. Packer, Mark J. Walker, Michael P. Jennings, Martina L. Sanderson-Smith

**Affiliations:** a School of Biological Sciences and Illawarra Health and Medical Research Institute, University of Wollongong, Wollongong, Australia; b Institute for Glycomics, Griffith University, Gold Coast, Australia; c Department of Chemistry and Biomolecular Sciences, Macquarie University, Sydney, Australia; d Australian Infectious Diseases Research Centre and School of Chemistry and Molecular Biosciences, University of Queensland, Brisbane, Australia; e Institute for Molecular Bioscience, University of Queensland, Brisbane, Australia; f Centre for Immunity, Infection and Inflammation, University of California, San Diego, California, USA; Lund University; University of Mississippi Medical Center

## Abstract

*Streptococcus pyogenes* (group A streptococcus [GAS]) is responsible for over 500,000 deaths worldwide each year. The highly virulent M1T1 GAS clone is one of the most frequently isolated serotypes from streptococcal pharyngitis and invasive disease. The oral epithelial tract is a niche highly abundant in glycosylated structures, particularly those of the ABO(H) blood group antigen family. Using a high-throughput approach, we determined that a strain representative of the globally disseminated M1T1 GAS clone 5448 interacts with numerous, structurally diverse glycans. Preeminent among GAS virulence factors is the surface-expressed M protein. M1 protein showed high affinity for several terminal galactose blood group antigen structures. Deletion mutagenesis shows that M1 protein mediates glycan binding via its B repeat domains. Association of M1T1 GAS with oral epithelial cells varied significantly as a result of phenotypic differences in blood group antigen expression, with significantly higher adherence to those cells expressing H antigen structures compared to cells expressing A, B, or AB antigen structures. These data suggest a novel mechanism for GAS attachment to host cells and propose a link between host blood group antigen expression and M1T1 GAS colonization.

## IMPORTANCE

There has been a resurgence in group A streptococcal (GAS) invasive disease, which has been paralleled by the emergence of the highly virulent M1T1 GAS clone. Intensive research has focused on mechanisms that contribute to the invasive nature of this serotype, while the mechanisms that contribute to host susceptibility to disease and bacterial colonization and persistence are still poorly understood. The M1T1 GAS clone is frequently isolated from the throat, an environment highly abundant in blood group antigen structures. This work examined the interaction of the M1 protein, the preeminent GAS surface protein, against a wide range of host-expressed glycan structures. Our data suggest that susceptibility to infection by GAS in the oral tract may correlate with phenotypic differences in host blood group antigen expression. Thus, variations in host blood group antigen expression may serve as a selective pressure contributing to the dissemination and overrepresentation of M1T1 GAS.

## INTRODUCTION

One of the major alloantigenic systems in humans is the ABO(H) blood group antigen system. In addition to expression on red blood cells, ABO(H) glycan structures are expressed in extracellular fluids, such as saliva, tears, and breast milk, and at the surface of oral epithelial cells (1–3). The human blood group antigen classification system is based on three glycan structures, A, B and H, with precursors lacto-*N*-tetraose (LNT; Gal-β-1,3-GlcNAc-β-1,3-Gal-β-1,4-Glc), lactosamine type 1 (Galβ1-3GlcNAc), and lactosamine type 2 (Galβ1-4GlcNAc) forming the major backbone of all three antigens (4). Host gene expression of α-1,2-fucosyltransferase enables synthesis of H antigen, which can be further transformed by *N*-acetylgalactosaminyl-transferase or d-galactosyl-transferase to produce A and B antigens, respectively (5, 6). Expression of A, B, and H antigens on epithelial cell surfaces and secretion into extracellular fluid is dependent on expression of the Se(*FUT2*) gene encoding fucosyl-transferase 2, which facilitates secretion of the corresponding blood group antigens by mucin-secreting goblet cells (1). Population studies have shown that on average, 76% of people are blood group antigen secretors and 24% are nonsecretors (7). Furthermore, frequency of secretor status is significantly higher among H-antigen-expressing individuals than non-H-antigen-expressing individuals (7). The global distribution of blood group antigen alleles varies significantly across demographic populations. The H antigen phenotype has the highest representation across the world, particularly in Western Europe and North America (8). Frequencies of blood type A antigen phenotypes are highest among Scandinavian and Eastern European populations, whereas typically low B antigen frequencies are comparatively higher in Indian populations (9–11).

ABO(H) antigens are expressed by multiple host tissue types. Primarily localized at the surface of epithelial cells, these glycans are thought to mediate contact with multiple viral and bacterial pathogens. Phenotypic differences in host glycan expression have been correlated to host disease susceptibility for several human bacterial pathogens, including *Helicobacter pylori*, *Campylobacter jejuni*, *Streptococcus pneumoniae*, *Salmonella enterica* serovar Typhimurium and *Staphylococcus aureus* (12–18). The first stage of bacterial infection is initiated by the specific recognition of host epithelial surfaces. Microorganisms often use sugar-binding proteins (lectins) to mediate this interaction. The human-specific pathogen group A streptococcus (GAS) typically colonizes epithelial cells of the skin and mucosal surfaces, where the bacteria encounter a diverse array of glycosylated structures, both attached to the cell surface and secreted in mucosal fluids. Despite this, interactions between GAS and host glycans remain poorly characterized. Due to technological advances in the field of glycobiology, the opportunity now exists to examine host-pathogen glycan interactions with increased efficiency and accuracy using glycan and lectin arrays (3, 14, 19). The M protein is the dominant protein at the surface of GAS and is thought to play a role in promoting adherence to host tissues (20–22). As the preeminent GAS cell surface protein, M protein is a strong candidate for mediating GAS-glycan interactions; however, to date, M protein-glycan interactions have not been comprehensively explored (23).

In the present work, we show that the globally disseminated M1T1 GAS clone binds multiple host blood group antigen structures via the surface-expressed M protein. Furthermore, we demonstrate that differences in M protein specificity for blood group antigens correlate with attachment to human oral epithelial cells. Blood group antigen structures and their related precursors are heavily represented on oral epithelia and in mucosal fluid, suggesting a role in GAS pharyngeal infection. These mechanisms of glycan interaction may contribute to the global dissemination of M1T1 GAS, particularly in Western populations.

## RESULTS

### Interaction of M1 protein with glycans.

**FIG 1:**
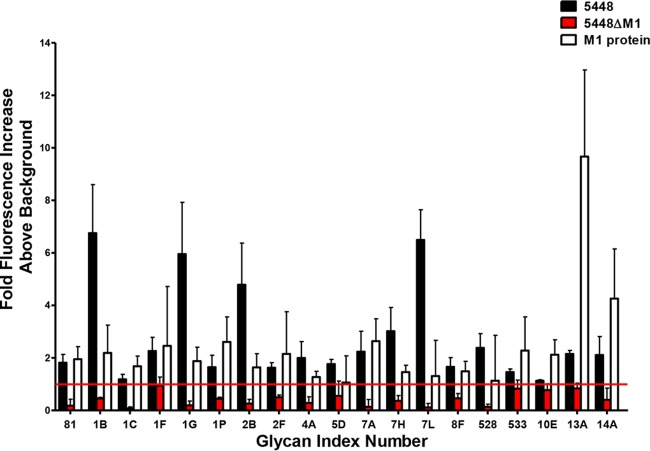
The M1 protein is a major lectin of M1T1 GAS clone 5448. Glycan binding of GAS strains 5448 and 5448ΔM1 along with recombinant M1 protein was analyzed using the ProScanArray imaging software ScanArray Express (PerkinElmer, USA), and the data were exported to Microsoft Excel for further analysis. Bacterial and protein binding to a glycan was defined as a value representing a >1-fold increase above the mean background relative fluorescence units (RFU). The mean background was calculated from the average RFU of all empty spots on the array + 3 standard deviations. Statistical analysis of the data was performed by a Student’s *t* test with a confidence level of 99.99% (*P* ≤ 0.0001), and only glycans that met these criteria for three biologically independent samples (*n* = 12 glycan spot replicates) were interpreted as positive binding interactions. Glycan index: 81, Galα1-4GlcNAcβ; 1B, Galβ1-4GlcNAc; 1C, Galβ1-4Gal; 1F, Galb1-3GalNAcb1-4Galb1-4Glc; 1G, Galβ1-3GlcNAcβ1-3Galβ1-4Glc; 1P, Galα1-3Galβ1-4Glc; 2B, Galβ1-6Gal; 2F, Galα1-4Galβ1-4GlcNAc; 4A, GlcNAcβ1-4GlcNAc; 5D, Manα1-3Man; 7A, Fucα1-2Galβ1-3GlcNAcβ1-3Galβ1-4Glc; 7H, Galβ1-4(Fucα1-3)Glc; 7L, Fucα1-2Galβ1-4(Fucα1-3)Glc; 8F, Galβ1-4(Fucα1-3)GlcNAcβ1-6(Fucα1-2Galβ1-3(Fucα1-4)GlcNAcβ1-3)Galβ1-4Glc; 528, Neu5Acα2-3Galβ1-4(Fucα1-3)GlcNAcβ1-3Galβ; 533, GalNAcβ1-4(Neu5Acα2-8)_2_Neu5Acα2-3Galβ1-4Glc; 10E, Neu5Acα2-3Galβ1-3(Neu5Acα2-6)GalNAc; 13A, ΔUA 2S-GalNAc-4S Na_2_ (Δ Di-disB); 14A, (GlcAβ1-3GlcNAcβ1-4)*n* (*n* = 10).

To assess the diversity of GAS-glycan interactions, glycan microarray analysis was undertaken using wild-type (WT) M1T1 GAS clone 5448, isogenic mutant 5448ΔM1, and recombinant M1 protein. Of the 358 glycan structures present on the array, GAS strain 5448 was observed to bind 204 distinct structures. In particular, binding was observed between strain 5448 and multiple terminal galactose, terminal *N*-acetylglucosamine, mannose-containing, Neu5Ac-containing, fucosylated, and glycosaminoglycan structures (see Table S1 in the supplemental material). M protein is a major surface protein and virulence factor of GAS, contributing to multiple stages of disease (20, 24). We therefore assessed whether GAS 5448 glycan binding could be mediated via its surface M1 protein. Glycan microarray analysis was undertaken using both recombinant M1 protein and the isogenic M1 protein-deficient GAS mutant 5448ΔM1. M1-dependent glycan interactions were assigned to structures recognized by both WT GAS 5448 and the recombinant M1 protein, but not the isogenic mutant strain 5448ΔM1. Of the 204 glycan structures bound by GAS strain 5448, 19 bound in an M1 protein-dependent fashion, including blood group antigen precursor LNT (glycan index no. 1G) and blood group antigen H type I (glycan index no. 7A) (Fig. 1). In addition, both 5448 and 5448ΔM1 bound multiple blood group A (glycan index no. 7K, 235, 366, 368, and 392) and B (glycan index no. 7M, 226, 359, 306, 362, and 363) antigen species. These data suggest the presence of additional lectin receptors at the GAS cell surface (Table S1). As blood group antigen structures are commonly expressed on human tissue and in mucosal fluid, the interactions of M1 with both the H antigen type I and the structurally similar LNT were selected for more detailed characterization (1, 3).

10.1128/mBio.02237-16.1 TABLE S1  Recombinant M1 protein, GAS strain 5448, and 5448ΔM1 glycan binding profile. Glycan binding was analyzed using the ProScanArray imaging software ScanArray Express (PerkinElmer, USA), and the data were exported to Microsoft Excel for further analysis. M1 protein binding to a glycan was defined as a value representing a ≥1-fold increase above mean background RFU. The mean background was calculated from the average RFU of all empty spots on the array + 3 standard deviations. Furthermore, statistical analysis of the data was performed by a Student’s *t* test with a confidence level of 99.99% (*P* ≤ 0.0001), and only glycans that met these criteria for three biologically independent samples (*n* = 12 glycan spot replicates) were interpreted as positive binding interactions (highlighted green). Download Table S1, PDF file, 0.6 MB. Copyright © 2017 De Oliveira et al. 2017 De Oliveira et al. This content is distributed under the terms of the Creative Commons Attribution 4.0 International license.

### M1 protein is a high-affinity blood group antigen receptor.

**FIG 2:**
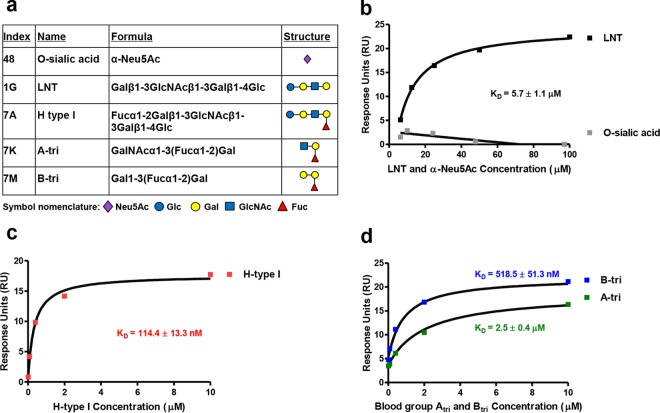
M1 protein binds blood group antigen-related structures with various affinities. (a) Structural comparison of glycans used in SPR binding experiments. Index numbers correspond to glycan index numbers used in Table S1. M1 binding to (b) lacto-*N*-tetraose (LNT; Galβ1-3GlcNAcβ1-3Galβ1-4Glc), negative binding control α-Neu5Ac, (c) H antigen type I (Fucα1-2Galβ1-3GlcNAcβ1-3Galβ1-4Glc), and (d) blood group A trisaccharide [A-tri; GalNAcα1-3(Fucα1-2)Gal] and blood group B trisaccharide [B-tri; Galα1-3(Fucα1-2)Gal] was determined via SPR and modeled using steady-state affinity fitting of the single-cycle kinetic sensorgrams. Affinity constants for the binding interactions are provided in the figure panels.

To further characterize the interaction of M1 protein with LNT and H antigen type I, single-cycle kinetic surface plasmon resonance (SPR) analysis was undertaken using a Biacore T200. In addition, the interaction between M1 and blood group A [types I and II; GalNAcα1-3(Fucα1-2)Gal] and B [types I to IV; Galα1-3(Fucα1-2)Gal] antigen trisaccharides was assessed (Fig. 2a). SPR revealed that M1 protein bound LNT with an affinity of 5.7 ± 1.1 µM, confirming the results of the glycan microarray (Fig. 2b). The binding of M1 to mature blood group antigen structures was significantly stronger than binding to LNT, with M1 showing the highest affinity for the H antigen type I structure (equilibrium dissociation constant [*K*_*D*_] = 114.4 ± 13.3 nM) compared with B antigen (*K*_*D*_ = 518.5 ± 51.3 nM) and A antigen (*K*_*D*_ = 2.5 ± 0.4 µM) structures (Fig. 2c and d). Increased affinity for blood group antigen structures compared with LNT suggests a role for fucose residues in high-affinity M1 protein-glycan interactions. No interaction was seen between M1 and the negative-control glycan *O*-sialic acid (α-Neu5Ac; glycan index no. 48) (Fig. 2a and b). Furthermore, M98 from skin-tropic GAS isolate NS88.2 was unable to bind respective blood group A, B, and H antigen-type structures (data not shown).

### M1 protein preferentially binds blood group antigen H.

**FIG 3:**
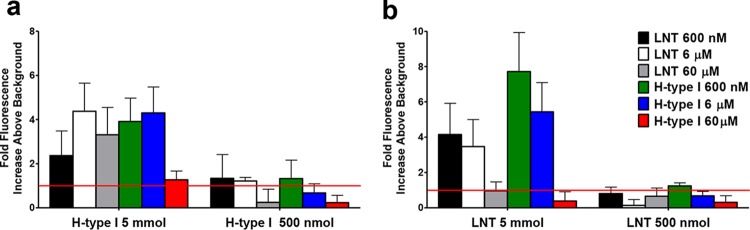
H antigen type I and LNT binding are localized to the same site in M1 protein. M1 binding to immobilized (a) H antigen type I (Fucα1-2Galβ1-3GlcNAcβ1-3Galβ1-4Glc) and (b) lacto-*N*-tetraose (LNT; Galβ1-3GlcNAcβ1-3Galβ1-4Glc) was assessed in the presence of various concentrations of H antigen type I and LNT. Glycan binding was analyzed using the ProScanArray imaging software ScanArray Express (PerkinElmer, USA), and the data were exported to Microsoft Excel for further analysis. M1 protein binding to a glycan was defined as a value representing a >1-fold increase above mean background RFU. The mean background was calculated from the average RFU of all empty spots on the array + 3 standard deviations. Statistical analysis of the data was performed by a Student’s *t* test with a confidence level of 99.99% (*P* ≤ 0.0001), and only glycans that met these criteria for three biologically independent samples (*n* = 12 glycan spot replicates) were interpreted as positive binding interactions.

LNT is a major precursor of H antigen type I synthesis. Both glycan species are highly similar in structure, differing only in α1-2-linked fucosylation at the terminal galactose residue (Fig. 2a). To assess whether a common binding site for these two glycan structures exists within M1 protein, we performed competitive glycan array analysis using both LNT and H antigen type I. Competition binding analysis was done by preincubating recombinant M1 protein with various concentrations of either LNT or H antigen type I (600 nM to 60 µM) prior to determining the binding interaction with the respective immobilized glycans. Preincubation of M1 with LNT was shown to reduce binding to 500 nmol immobilized H antigen type I (Fig. 3a). Similarly, preincubation of M1 with H antigen type I reduced binding to 500 nmol and 5 mmol amounts of immobilized LNT (Fig. 3b). These data suggest that LNT and H antigen type I bind to the same site within the M1 protein. However, preincubation of M1 with LNT did not prevent binding of M1 to the largest amount of immobilized H antigen type I (5 mmol) (Fig. 3a). Additionally, preincubation of M1 with 60 µM H antigen type I inhibited binding to the highest molar amount of immobilized LNT (5 mmol) (Fig. 3b). These data confirm that M1 has a higher affinity for H antigen type I than for LNT.

### M1 protein mediates glycan binding via the B repeat domains.

**FIG 4:**
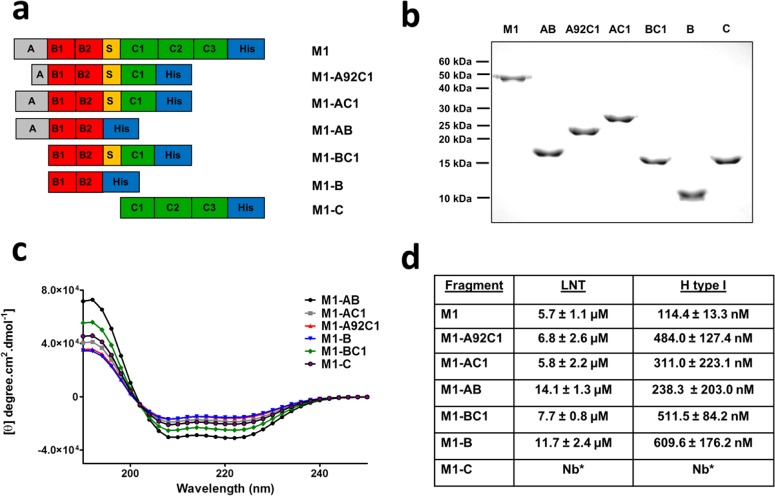
Binding of LNT and H antigen type I is localized to the B repeat domains of M1. (a and b) Full-length M1 and M1 fragment constructs expressing overlapping repeat domains, ranging from 51 to 9 kDa. (c) CD spectra of M1 and M1 fragment constructs. All recombinant M1 proteins exhibited CD emission spectra consistent with α-helical coiled-coil proteins. (d) SPR binding analysis. Binding of LNT and H antigen type I was found to be localized to the B repeat domains of M1. Binding was modeled using steady-state affinity fitting of the single-cycle kinetic sensorgrams.

To determine which domains of M1 are involved in binding to LNT and H antigen type I, truncated M1 protein fragments with overlapping repeat domains were constructed as previously described (25, 26) (Fig. 4a). As predicted, the apparent molecular masses of the M1 fragments ranged from 27 kDa to 9 kDa (Fig. 4b) with far-UV circular dichroism (CD) spectra characteristic of α-helical coiled-coil proteins (Fig. 4c). SPR binding analysis of each M1 fragment demonstrated that, with the exception of fragment M1-C, all M1 protein fragments bound LNT (*K*_*D*_ = 6.8 to 14.1 µM) and H antigen type I (*K*_*d*_ = 238.3 to 609.6 nM) (Fig. 4d). These data, in conjunction with the competition glycan array analysis (Fig. 3a and b), suggest the existence of a galactose glycan-binding site or sites localized to the B repeat domains of M1.

**FIG 5:**
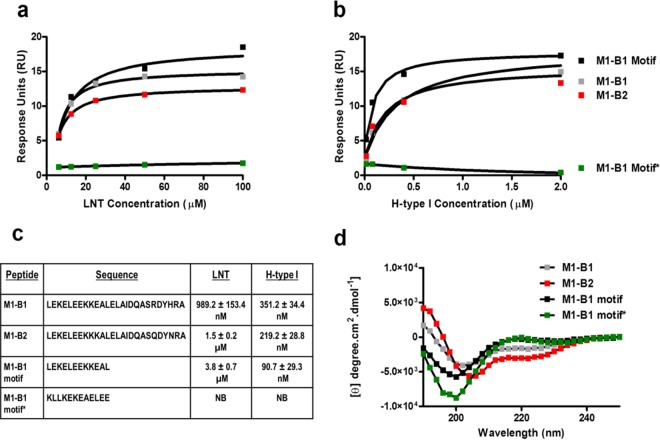
Both B repeat domains of M1 can bind LNT and H antigen type I. Binding analysis of the M1-derived peptides M1 B1, M1 B2, M1 B1 motif, and scrambled M1 B1 motif* were screened for binding against (a) lacto-*N*-tetraose (LNT; Galβ1-3GlcNAcβ1-3Galβ1-4Glc) and (b) H antigen type I (Fucα1-2Galβ1-3GlcNAcβ1-3Galβ1-4Glc). Binding was analyzed via SPR and was modeled using steady-state affinity fitting of the single-cycle kinetic sensograms. (c) Dissociation constants of LNT and H type I binding interaction with M1 B1, M1 B2, M1 B1 motif, and scrambled M1 B1 motif* peptides. (d) CD spectra of M1-derived peptides. The M1 B1, M1 B2, M1 B1 motif, and M1 B1 motif* peptides exhibited CD emission spectra consistent with that of a random coil (30).

To ascertain whether individual B repeat domains of M1 protein bind blood group-related glycan structures, SPR binding analysis was performed using peptides corresponding to the B1 and B2 repeat domains of M1. Binding analysis revealed that both B1 and B2 peptides were able to bind LNT (M1 B1, *K*_*D*_ = 989.2 ± 153.4 nM; M1 B2, *K*_*D*_ = 1.5 ± 0.2 μM) and H antigen type I (M1 B1, *K*_*D*_ = 351.2 ± 34.4 nM; M1 B2, *K*_*D*_ = 219.2 ± 28.8 nM) with various affinities (Fig. 5a to c). The glycan binding motif within the M1 protein was further refined using a peptide representing the first 12 amino acids of the B1 repeat domain and sharing 92% sequence identity with the B2 repeat (M1 B1 motif: LEKELEEKKEAL_HHHHHHHHHH_). SPR demonstrated that the M1 B1 motif peptide exhibited binding to LNT (*K*_*D*_ = 3.8 ± 0.7 μM) and H antigen type I (*K*_*D*_ = 90.7 ± 29.3 nM). A scrambled adaptation of the M1 B1 motif sequence (M1 B1 motif*; KLLKEKEAELEE_HHHHHHHHHH_) did not recognize LNT or H antigen type I (Fig. 5a to c). These data indicate that the structure adopted by these 12 amino acids within the M1 B repeat domain is critical for recognition of specific blood group-related antigen structures. Secondary structure analysis using far-UV CD showed that M1 B1 and M1 B2 peptides exhibited a prominent negative band at 208 nm, indicative of peptides with both α-helical and random-coil contents (27). The M1 B1 motif and M1 B1 motif* peptides adopted random coil-like structures (28) (Fig. 5d). Thus, binding of LNT and H antigen type I may in fact involve a specific, nonhelical conformation of the M1 B repeat domain.

### Differences in host blood group antigen expression affect GAS association with oral epithelial cells.

Pharyngitis is most frequently attributed to a subset of GAS M types, including serotypes M12, M3, and M1 (20, 21). The epithelial cells lining the oral mucosa provide a glycan-rich environment, expressing a multitude of O- and N-linked carbohydrate structures (3, 29). To explore the potential relationship between blood group antigen expression and GAS association with epithelial cells of the oral mucosa, the ability of enhanced green fluorescent protein-expressing strain 5448 (5488eGFP) to associate with human buccal epithelial (HBE) cells was examined via flow cytometry. As previously shown by Everest-Dass et al. (3), salivary glycans reflect the secretor and ABO(H) blood group status similar to HBE cells. Therefore, to identify the secretor and blood group status of HBE cells from various donors, liquid chromatography-electrospray ionization mass spectrometry (LC-ESI MS) analysis of released O-linked glycans from salivary glycoproteins of 38 donors was carried out. Negative-ion MS^2^ fragmentation spectra were used for detailed structural analysis of the blood group antigen-determining structures (see Fig. S2 in the supplemental material). Of the 38 donors, 4 were observed to be blood group antigen nonsecretors. From the remaining 34 donors, 11 expressed the A antigen, 6 coexpressed A and B antigens, 9 expressed lone H antigen structures, and 8 were shown to express the B antigen moiety.

10.1128/mBio.02237-16.5 FIG. S1  Glycan binding profile of M1T1 clone GAS strains 5448 and 5448ΔM1 to blood group antigen A- and B-related structures. Glycan binding was analyzed using the ProScanArray imaging software ScanArray Express (PerkinElmer, USA), and the data were exported to Microsoft Excel for further analysis. Bacterial binding to a glycan was defined as a value representing a ≥1-fold increase above mean background RFU. The mean background was calculated from the average RFU of all empty spots on the array + 3 standard deviations. Statistical analysis of the data was performed by a Student’s *t* test with a confidence level of 99.99% (*P* ≤ 0.0001), and only glycans that met these criteria for three biologically independent samples (*n* = 12 glycan spot replicates) were interpreted as positive binding interactions. Glycan index: 226, Galα1-3(Fucα1-2)Galβ; 359, Galα1-3(Fucα1-2)Galβ1-3GlcNAcβ; 360, Galα1-3(Fucα1-2)Galβ1-4GlcNAcβ; 362, Galα1-3(Fucα1-2)Galβ1-3GalNAcα; 363, Galα1-3(Fucα1-2)Galβ1-3GalNAcβ; 368, GalNAcα1-3(Fucα1-2)Galβ1-4GlcNAcβ; 7K, GalNAcα1-3(Fucα1-2)Gal. Download Fig. S1, PDF file, 0.2 MB. Copyright © 2017 De Oliveira et al. 2017 De Oliveira et al. This content is distributed under the terms of the Creative Commons Attribution 4.0 International license.

10.1128/mBio.02237-16.2 FIG. S2  Representative mass spectra of *O*-glycans released from salivary glycoprotein proteins. Shown are collision-induced dissociation-tandem MS (MS/MS) fragment spectra of singly charged negative ions of three O-linked glycan structures from saliva at (a) *m/z* 530.3^1−^, (b) *m/z* 733.3^1−^ and (c) *m/z* 895.3^1−^. The fragmentation spectra of these unique glycan species represented here were used to identify secretor and ABO(H) blood group status. For example, the structure with *m/z* 530.3^1−^ is indicative of an H antigen and was observed to be present only in secretor individuals. Similarly, the fragmentation spectra of *m/z* 733.3^1−^ show an *O*-glycan structure with blood group A epitope and *m/z* 895.3^1−^ with blood group B epitope. Download Fig. S2, PDF file, 0.2 MB. Copyright © 2017 De Oliveira et al. 2017 De Oliveira et al. This content is distributed under the terms of the Creative Commons Attribution 4.0 International license.

**FIG 6:**
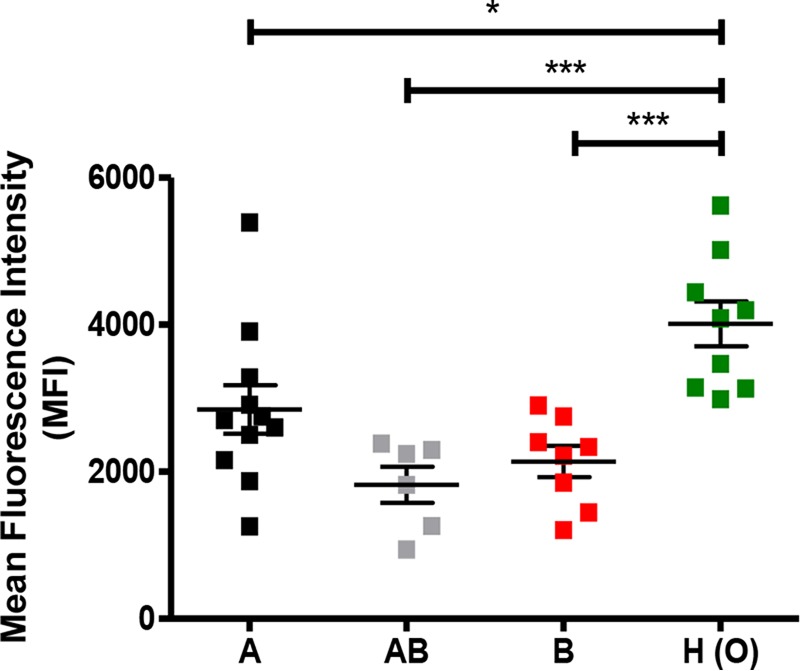
Blood group antigen interactions with M1T1 GAS strain 5448 promote association with HBE cells. Association of the 5448 WT to HBE cells expressing A, B, A and B, or H blood group antigen structures was examined using flow cytometric analysis.

HBE cells were identified by their characteristic forward and side scatter profiles, and a single gate was used to exclude debris and nonassociated GAS (3) (see Fig. S3 in the supplemental material). Flow cytometry analysis demonstrated that 5448eGFP was able to associate with HBE cells expressing H antigen structures at a significantly higher level than cells expressing A (*P* < 0.05), AB (*P* < 0.001), or B antigen structures (*P* < 0.001) (Fig. 6).

10.1128/mBio.02237-16.3 FIG. S3  Flow cytometry assay of WT 5448 binding to human buccal epithelial cells. Dot plots at the top of each panel show the forward scatter (FSC-A) and the side scatter (SSC-A) distribution, while the corresponding histograms of buccal epithelial cell count versus fluorescence intensity at 515 to 520 nm are shown below. (a) Autofluorescence of buccal epithelial cells alone. (b) WT 5448eGFP incubated with buccal epithelial cells. Download Fig. S3, PDF file, 0.2 MB. Copyright © 2017 De Oliveira et al. 2017 De Oliveira et al. This content is distributed under the terms of the Creative Commons Attribution 4.0 International license.

To assess the importance of M1 B repeat domains and M1 B repeat coiled-coil irregularities in blood group antigen recognition, quantitative HBE adherence and internalization assays were undertaken using the 5448 WT and 5448M1* (which expresses “idealized” α-helical M1 B repeat domains). Replacement of alanine amino acids responsible for the irregularities of the coiled-coil structure in the B repeat domains has been used to generate the “idealized” coiled-coil version of the M1 protein in 5448M1*. Stabilization of the M1 B repeat domains has been previously shown to attenuate binding function (25, 30).

**FIG 7:**
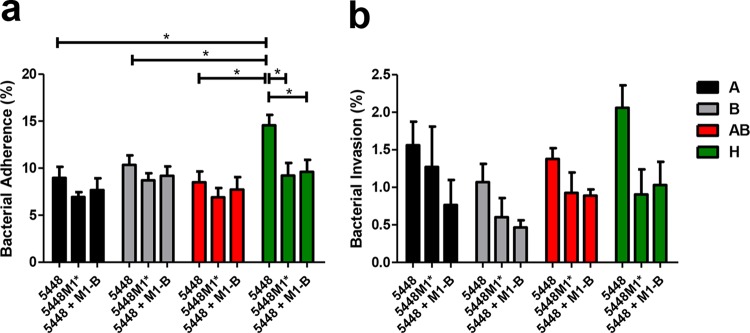
M1T1 GAS strain 5448 preferentially adheres to and invades H-antigen-expressing HBE cells via the M1 protein B repeat domains. (a) Adherence and (b) invasion of HBE cells by M1T1 GAS strains 5448 and 5448M1* in the presence and absence of 100 μM M1 B repeat fragment. Data are representative of three independent experiments performed in triplicate. Adherent and internalized bacteria were normalized to the inoculum.

GAS strain 5448 was observed to adhere to the surface of H-antigen-expressing HBE cells at a significantly higher level than HBE cells expressing A (*P* < 0.05), B (*P* < 0.05), or both A and B moieties (*P* < 0.05) (Fig. 7a). In the presence of exogenous M1 B fragment, adherence of 5448 to H-antigen-expressing HBE cells was significantly reduced (*P* < 0.05), demonstrating comparative levels of adherence to that of 5448M1* (*P* < 0.05) (Fig. 7a). No significant differences in 5448 internalization were observed across HBE cells of different blood group antigen status. Although differences in internalization were not statistically significant, the highest level of 5448 invasion was observed with H antigen-expressing HBE cells. Internalization of 5448M1* and 5448 in the presence of exogenous M1 B fragment decreased relative to the 5448 WT across all HBE blood group antigen phenotypes (Fig. 7b). These data suggest that both the presence and the disordered α-helical structure of the M1 B repeat domains are crucial in blood group antigen recognition by M1T1 GAS.

## DISCUSSION

Since the 1980s, there has been a marked resurgence in invasive GAS disease paralleled by the emergence of the highly virulent M1T1 GAS clone (31–33). Representation of M1 serotypes is highest in urbanized Western areas such as Western Europe and the Americas (34–38). Epidemiological studies have identified that M1T1 GAS clones are frequently isolated from throat cultures, representing a common cause of pharyngitis, and this may contribute to the overrepresentation of these isolates in more severe infections (39). The pharyngeal tract is glycan-rich, and bacterium-glycan interactions are critical in host colonization for several pathogens. To date, the characterization of GAS-glycan interactions has focused on large glycosaminoglycans such as heparin, heparin sulfate, and dermatan sulfate (23, 40). Recent studies analyzing the hemolytic GAS pore-forming cytolysin streptolysin O (SLO) identified that SLO interaction with lacto-*N*-neotetraose on red blood cells facilitates hemolytic activity (41). Although SLO is secreted and not expressed at the GAS cell surface, this protein exemplifies one of the few characterized GAS lectins. Glycans are highly diverse in both structure and function, and decoding bacterial interactions with a wide range of glycan structures has proved challenging. Here, we have systematically characterized novel M1 protein-glycan interactions that may contribute to the selective persistence of M1T1 serotype GAS in the upper respiratory tract.

Blood group antigen structures are expressed by a range of host cells (2, 3). It is well established that GAS adheres to epithelial cells of the skin and pharyngeal mucosa (20, 42). Previous studies analyzing *emm* pattern E (generalist) M28 and *emm* pattern A-C (throat-tropic) M6 GAS suggest that M protein interaction with fucose and galactose, essential components of blood group antigens, plays an important role in GAS adherence to the host pharyngeal mucosa (43, 44). Furthermore, we have shown that M protein from *emm* pattern D (skin-tropic) M98 was unable to bind blood group antigen structures. Therefore, we propose that interaction with blood group antigen structures may be a function of throat-tropic GAS. Here, we found that GAS strain 5448 interacts with the blood group antigen precursor LNT and H antigen type I in an M1 protein-dependent manner. Several blood group A and B antigen structures were also found to bind purified recombinant M1 and M1T1 GAS strain 5448 and also interacted with 5448ΔM1, suggesting that in addition to M1, GAS express other lectins with blood group antigen specificity. In the present study, LNT and H antigen type I interactions were shown to be restricted to a 12-amino-acid sequence (LEKELEEKKE/KAL) present at the start of both the B1 and B2 repeat domains of M1. In the reported crystal structure of the dimeric AB fragment of M1, the B repeat regions are splayed apart (see Fig. S4a in the supplemental material), and although they are in a helical conformation due to anti-parallel coiled-coil formation with a neighboring M1 molecule in the crystal (Fig. S4b), complementary far-UV CD analyses suggest the B repeat domains have a less helical structure in solution (25), which is consistent with our CD analyses of M1 B repeat peptides. As such, the lectin domain identified here may adopt a flexible structure within the M1 protein, which is able to recognize various galactose-containing carbohydrates distinct in structure and function.

10.1128/mBio.02237-16.4 FIG. S4  (a) Crystal structure of the dimeric AB fragment of M1 (Protein Data Bank ID 2OTO [20]) (b). Antiparallel coiled-coil interactions between two M1AB dimers (dark and light teal) in the crystal. The minimal motif shown to bind LNT and H antigen type 1 is highlighted in orange. The structures are shown in cartoon representation as well as surface representation in panel a. The figure was prepared using PyMol (Schrödinger, LLC). Download Fig. S4, PDF file, 0.2 MB. Copyright © 2017 De Oliveira et al. 2017 De Oliveira et al. This content is distributed under the terms of the Creative Commons Attribution 4.0 International license.

Fucosylation of LNT, which results in blood group H antigen type I, significantly increased the affinity of M1 for LNT. Similarly, M1 protein had a significantly higher affinity for blood group A and B trisaccharides, which both contain α2-linked fucose residues, than for LNT. Studies analyzing the blood group recognition properties of rotaviruses show that fucose moieties do not play a direct role in blood group antigen binding (45). Rather, it has been hypothesized that fucose residues play an indirect role by stabilizing the blood group antigen via nonconventional hydrogen bonding (13). Although the lectin binding mechanisms of M1 are yet to be fully elucidated, we speculate that fucose is not essential for recognition of blood group-related structures but instead mediates a higher-affinity interaction with the B repeats of M1, which may parallel the observed lectin properties of both rotaviruses and noroviruses (13, 45).

The interaction of M1 protein with fibrinogen both protects GAS against phagocytosis and stimulates neutrophil activation via activation of a proinflammatory cascade (26, 42, 46). The M1-fibrinogen binding interaction is mediated by the B repeat domains of M1, and a fibrinogen-binding motif within the B repeat domain has been predicted (21, 26). This predicted binding motif is located directly downstream of the site proposed to mediate blood group antigen binding in the present study. It is possible that fibrinogen may sterically hinder M1 blood group antigen interaction. Alternatively, the small molecular mass of blood group antigen structures relative to fibrinogen, the presence of independent binding motifs, and the low concentration of fibrinogen in saliva could facilitate the colocalization of both fibrinogen and blood group antigens to the M1 protein and GAS cell surface.

M1T1 GAS are commonly isolated from the upper respiratory tract during the course of pharyngeal infection (39). Here, we show that association of strain 5448 with HBE cells was significantly increased when H antigen structures were expressed at the epithelial cell surface. While differences in the expression profiles of host cell surface proteins such as Toll-like receptors, integrins, and donor variations in mucin secretions cannot be discounted, our data suggest that variations in cell surface glycan profile may contribute to differences in individual susceptibility to GAS colonization and disease. Although multiple factors are involved, interaction of M1T1 GAS with blood group antigen and related structures may underpin mechanisms of global dissemination and transmission.

The globally disseminated M1T1 clone of GAS is a significant cause of human disease. Here, we have identified a range of novel M1 protein-dependent glycan interactions, suggesting a role for galactose-containing structures in GAS colonization of the upper respiratory tract. Furthermore, we have provided evidence to support a potential link between host blood group antigen expression and GAS colonization status. This study exemplifies the diverse nature of lectin-glycan binding in the human host-GAS pathogen interaction.

## MATERIALS AND METHODS

### Bacterial strains, culture, and expression conditions.

*Escherichia coli* strains TOP10 and BL21(DE3), containing pGEX2T and pet-28b(+) expression plasmids, were cultured and used for expression of recombinant proteins at 37°C in lysogeny broth supplemented with ampicillin (100 µg/ml) or kanamycin (50 µg/ml), as previously described (25, 47). M1T1 GAS clone 5448, isogenic mutant 5448ΔM1, and 5448M1* expressing “idealized” M1 protein B repeat domains have also been described previously (30, 48, 49). Bacteria were transformed with enhanced green fluorescent protein (eGFP)-expressing plasmid as described by Ly et al. (50). GAS isolates were cultured overnight at 37°C on horse blood agar (BioMérieux, Sydney, Australia) or in static liquid cultures of Todd-Hewitt broth (BD, Sydney, Australia) supplemented with 1% (wt/vol) yeast extract (THY medium [Oxoid, Adelaide, Australia]). Peptides were purchased from GenScript (Piscataway, NJ), and included a C-terminal deca-histidine tag.

### Glycan microarray analysis.

A glycan microarray was employed to identify novel GAS-glycan interactions (14). Whole-cell GAS glycan array analysis was undertaken using M1T1 GAS strain 5448 and the isogenic mutant 5448ΔM1. GAS strains were harvested at an optical density at 600 nm (OD_600_) of 0.5 by centrifugation (5,000 × *g*, 10 min), and 1 × 10^8^ CFU were resuspended in array phosphate-buffered saline (PBS [PBS with 2 mM MgCl_2_ and 2 mM CaCl_2_]) containing 1 mM Cell Trace BODIPY TR methyl ester (Thermo Fisher Scientific, Australia), to a final volume of 250 μl. Staining of bacteria was allowed to proceed in the dark at room temperature for 15 min. Following staining, bacterial cells were passed through a 5 μm-pore Millex-SV syringe filter unit (Merk-Millipore, Australia) to remove clumps of aggregated bacteria. For glycan array analysis of recombinant M1 protein, purified M protein (1 μg) was preincubated with mouse anti-His tag antibody (Cell Signalling, Australia), rabbit anti-mouse Alexa Fluor 488 antibody conjugate (Thermo Fisher Scientific, Australia) and goat anti-rabbit Alexa Fluor 488 conjugate (Thermo Fisher Scientific, Australia), at a ratio of 4:2:1. For competitive glycan binding analysis, M protein-antibody complexes were preincubated with glycans of interest (600 nM to 60 μM) purchased from Dextra Laboratories (United Kingdom). Protein mixtures were made up to a final volume of 65 μl in array PBS and incubated at room temperature for 10 min to allow protein complex formation. Glycan microarray slides were blocked in array PBS containing 0.1% (wt/vol) bovine serum albumin (Sigma-Aldrich, Australia) for 10 min at room temperature and dried by centrifugation (200 × *g*, 5 min). Following centrifugation, gene frames (ABgene, Australia) were bonded onto each glycan microarray slide, and 65 μl of GAS or precomplexed protein mixture was applied. Mixtures were secured with the addition of a plastic coverslip, and samples were allowed to hybridize in the dark for 10 min at room temperature. Proceeding incubation, glycan microarray slides were washed in array PBS for 5 min with agitation, followed by a secondary wash in array PBS containing 0.001% Tween 20 for 2 min with agitation. After washing, glycan microarray slides were dried by centrifugation (200 × *g*, 5 min). Fluorescence intensity of array spots was measured using the ProScanArray microarray 4-Laser scanner (PerkinElmer, USA) with the blue argon 488-nm excitation laser set to the fluorescein isothiocyanate (FITC) setting (494-nm excitation and 518-nm emission) and gain set to 40 to 60%. Image analysis was carried out using the ProScanArray imaging software ScanArray Express (PerkinElmer, USA). Positive binding was defined as greater than a 1-fold increase of the mean background + 3 standard deviations. Statistical analysis of the data was performed using a Student’s *t* test with a confidence level of 99.99% (*P* ≤ 0.0001), and only glycans that met these criteria for three biologically independent samples (*n* = 12 glycan spot replicates) were interpreted as positive binding interactions.

### SPR.

The interaction of M1 protein, M1 protein fragments and M1 peptides with specific glycan subsets was further investigated via single-cycle kinetic surface plasmon resonance (SPR) on a series S nitrilotriacetic acid (NTA) chip (Biacore AB) using a Biacore T200 (GE Healthcare, Sweden) at 25°C. The surface of the NTA chip was prepared by first stripping the surface with a regeneration buffer (0.35 M EDTA, 0.15 M NaCl, 0.01 M sodium phosphate buffer [pH 8.3]) for 1 min at 10 µl/min. The surface was then activated by an injection of 500 µM NiCl_2_ for 1 min at 10 µl/min. Recombinant proteins were captured via the C-terminal histidine tag on flow cells 2, 3, and 4 for 120 s at 10 µl/min. Flow cell 1 served as a reference cell, whereby a negative-control his-tagged protein was immobilized. Glycan analytes were diluted in PBS, and binding assays were performed using various concentrations of analyte (0 to 100 μM) over a series of five 120 s injections at a flow rate of 30 μl/min. Regeneration of the flow cell surface was achieved with two separate injections of the EDTA regeneration buffer for 60 s at 30 μl/min. M protein-glycan interactions were analyzed via a steady-state affinity model using Biacore T200 evaluation software (Biacore AB).

### Collection of saliva and human buccal epithelial cells.

Human buccal epithelial (HBE) cells were used to assess the role of blood group antigens in GAS attachment to host cells. Blood group status of 38 donors was determined via mass spectrometry of saliva samples from each donor. Unstimulated saliva and HBE cells were collected and prepared as previously described (3).

### Mass spectrometry.

Phenotypic differences in blood group antigen expression and secretory status of all 38 donors was characterized via electrospray ionization mass spectrometry of released salivary O-linked glycans as previously described (3).

### Association and internalization of GAS to HBE cells.

To assess the association of GAS to HBE cells, 2 × 10^5^ HBE cells were incubated with 2 × 10^6^ 5448eGFP cells in 250 μl Dulbecco’s PBS (DPBS) for 2 h at 37°C with 5% CO_2_. Postincubation, samples were resuspended in 750 μl DPBS, pelleted by centrifugation (500 × *g*, 10 min), and resuspended in a further 500 μl DPBS. Association of 5448eGFP with HBE cells was measured using an LSR II (BD Bioscience, USA) flow cytometer. Gated HBE cells were analyzed with a 575/26-nm filter, with data being recorded for 10,000 events. Fluorescence intensity of uninfected HBE cells was also measured to allow for compensation of autofluorescence. The relative quantity of 5448eGFP associated with HBE cells was estimated by the mean fluorescence intensity (MFI) of eGFP-positive HBE cells. Data were analyzed and processed using FlowJo (Treestar, USA).

To examine levels of bacterial internalization, HBE cells were incubated with either 5448 or 5448M1* as described above. Alternatively, 5448 was also incubated with HBE cells in the presence of M1 B fragment (100 µM). To kill nonadherent and HBE cell surface-adhered GAS, samples were treated with gentamicin (100 µg/ml) for 1 h at 37°C with 5% CO_2_. HBE cells were then pelleted by centrifugation, washed three times in DPBS, and lysed via resuspension in 0.025% (vol/vol) Triton X-100 (Sigma-Aldrich, Australia). Internalized CFU were determined via enumeration on THY agar. To measure GAS adherence, HBE cells were incubated in the absence of gentamicin, and total adherence was determined by subtracting CFU of total internalized GAS. Adherence and internalization assays were undertaken in triplicate with HBE cells isolated from 3 different donors. Statistical analysis was determined via a one-way analysis of variance, with a Tukey’s post hoc test. Homogeneity of variances was confirmed using a Bartlett’s test for equal variances.

### Ethics for human tissue collection.

Collection of HBE cells and saliva was performed with the approval of the University of Wollongong Human Ethics Committee (HE08/250). Volunteers provided informed consent before donating samples.
