# Supplementary material for: Blood Group Antigen Recognition via the Group A Streptococcal M Protein Mediates Host Colonization
Source: mBio. 2017 Jan 24;8(1):e02237-16. doi: 10.1128/mBio.02237-16 (PMC5263248; doi:10.1128/mBio.02237-16)
Supplement: TABLE S1 [file mbo002173156st1.pdf]

Supplementary Table 1: Recombinant M1 protein, GAS strain 5448 and 5448ΔM1 glycan binding profile. Glycan binding was analysed using ProScanArray imaging software, ScanArray Express (PerkinElmer, USA) and the data exported to Microsoft Excel for further analysis. M1 protein binding to a glycan was defined as a value ≥ 1 fold increase above mean background RFU. The mean background was calculated from the average RFU of all empty spots on the array plus three standard deviations. Furthermore, statistical analysis of the data was performed by a Student's *t*-test with a confidence level of 99.99% ($p \leq 0.0001$) and only glycans that met these criteria for three biologically independent samples (n = 12 glycans spot replicates) were interpreted as positive binding interactions (highlighted green).

ID. No.	Glycan name	Glycan structure	M1	±	5448WT	±	5448Δ M1	±
1A.	Lacto-N-biose I	Galβ1-3GlcNAc	4.47	1.54	0.19	0.07	0.14	0.02
1B.	N-Acetyllactosamine	Galβ1-4GlcNAc	2.19	1.06	6.75	5.84	0.46	0.04
1C.	β1-4galactosyl-galactose	Galβ1-4Gal	1.68	0.39	0.18	0.19	0.08	0.06
1D.	β1-6galactosyl-N-acetylglucosamine	Galβ1-6GlcNAc	2.83	1.48	0.14	0.04	0.08	0.19
1E.	β1-3galactosyl-N-acetylgalactosamine	Galβ1-3GalNAc	1.69	0.67	0.22	0.05	0.18	0.01
1F.	Galβ1-3GalNAcβ1-4Galβ1-4Glc		2.46	0.3	2.26	2.02	0.94	0.73
1G	Lacto-N-tetrose	Galβ1-3GlcNAcβ1-3Galβ1-4Glc	1.88	0.53	5.95	1.97	0.2	0.16
1H	Lacto-N-neotetrose	Galβ1-4GlcNAcβ1-3Galβ1-4Glc	3.78	2.28	2.23	0.04	1.23	0.08
1I.	Lacto-N-neohexose	Galβ1-4GlcNAcβ1-6(Galβ1-4GlcNAcβ1-3)Galβ1-4Glc	0.53	0.25	0.32	0.02	0.28	0.06
1J.	Lacto-N-hexose	Galβ1-4GlcNAcβ1-6(Galβ1-3GlcNAcβ1-3)Galβ1-4Glc	1.44	0.34	0.61	0.11	0.51	0.13
1K.	Globotriose	Galα1-4Galβ1-4Glc	1.37	0.63	0.37	0.16	0.4	0.33
1L.	Tn Antigen	GalNAcα1-O-Ser	2.38	0.91	0.32	0.15	0.38	0.11
1M.	Galactosyl-Tn Antigen	Galb1-3GalNAcα1-O-Ser	1.26	0.53	0.32	0.03	0.32	0.07
1N.	α1-3 Galactobiose	Galα1-3Gal	1.42	0.2	0.31	0.12	0.35	0.11
1O.	Linear B-2 Trisaccharide	Galα1-3Galβ1-4GlcNAc	2.02	0.43	0.33	0.05	0.33	0.13
1P.	Linear B-6 Trisaccharide	Galα1-3Galβ1-4Glc	2.61	0.95	1.65	2.04	0.45	0.06
2A.	α1-3, β1-4, α1-3 Galactotetrose	Galα1-3Galβ1-4Galα1-3Gal	1.05	0.39	0.46	0.1	0.47	0.17
2B.	β1-6Galactobiose	Galβ1-6Gal	1.64	0.52	4.79	1.59	0.26	0.16
2C.	Terminal disaccharide of globotriose	GalNAcβ1-3Gal	1.31	0.2	0.69	0.46	0.88	0.22
2D.	Receptor for pili of *P. aeruginosa*	GalNAcβ1-4Gal	1.81	0.75	0.3	0.25	0.39	0.27
2E.	P1 Antigen	Galα1-4Galβ1-4GlcNAc	1.53	0.11	0.31	0.13	0.25	0.14
2F.	α-D-N-acetylgalactosaminyl-1-3Gal-β1-4Glc	GalNAcα1-3Galβ1-4Glc	2.15	1.61	1.63	1.19	0.5	0.09
2G.	iso-Lacto-N-octose	Galβ1-3GlcNAcβ1-3Galβ1-4GlcNAcβ1-6(Galβ1-3GlcNAcβ1-3)Galβ1-4Glc	4.57	0.58	0.49	0.14	0.38	0.11
4A.	*N,N'*-Diacetyl chitobiose	GlcNAcβ1-4GlcNAc	1.27	0.22	2	0.66	0.29	0.23

4B.	*N,N',N''*-Triacetyl chitotriose	GlcNAcβ1-4GlcNAcβ1-4GlcNAc	1.16	0.36	0.64	0.43	0.32	0.37
4C.	*N,N',N'',N'''*-Tetraacetyl chitotetrose	GlcNAcβ1-4GlcNAcβ1-4GlcNAcβ1-4GlcNAc	1.34	0.33	0.5	0.15	0.54	0.18
4D.	*N,N',N'',N''',N'''',N'''''* -Hexaacetyl chitohexose	GlcNAcβ1-4GlcNAcβ1-4GlcNAcβ1-4GlcNAcβ1-4GlcNAcβ1-4GlcNAc	1.42	0.19	0.33	0.25	0.44	0.15
4E.	Bacterial cell wall muramyl discaccharide	GlcNAcβ1-4MurNAc	1.64	1.18	0.29	0.03	0.29	0.03
5A.	β1-2-*N*-Acetylglucosamine-mannose	GlcNAcβ1-2Man	2.07	1.08	0.22	0.05	0.18	0.04
5B.	Bianntennary *N*-linked core pentasaccharide	GlcNAcβ1-2Manα1-6(GlcNAcβ1-2Manα1-3)Man	2.27	0.76	0.44	0.08	0.44	0.18
5C.	α1-2-Mannobiose	Manα1-2Man	1.5	0.56	0.15	0.06	0.1	0.05
5D.	α1-3-Mannobiose	Manα1-3Man	1.06	1.02	0.77	0.18	0.55	0.56
5E.	α1-4-Mannobiose	Manα1-4Man	1.3	0.09	0.18	0.16	0.07	0.12
5F.	α1-6-Mannobiose	Manα1-6Man	0.76	0.21	0.34	0.09	0.39	0.06
5G.	α1-3, a1-6-Mannobiose	Manα1-6(Manα1-3)Man	1.99	1.12	0.3	0.05	0.28	0.22
5H.	α1-3, α1-3, α1-6-Mannopentaose	Manα1-6(Manα1-3)Manα1-6(Manα1-3)Man	1.74	0.62	0.13	0	0.11	0.11
7A.	Lacto-N-fucopentose I	Fucα1-2Galβ1-3GlcNAcβ1-3Galβ1-4Glc	2.64	0.83	2.24	1.78	0.14	0.28
7B.	Lacto-N-fucopentose II	Galβ1-3(Fucα1-4)GlcNAcβ1-3Galβ1-4Glc	1.37	0.2	0.22	0.1	0.15	0.01
7C.	Lacto-N-fucopentose III	Galβ1-4(Fucα1-3)GlcNAcβ1-3Galβ1-4Glc	1.42	0.99	0.2	0.06	0.19	0.25
7D.	Lacto-N-difucohexose I	Fucα1-2Galβ1-3(Fucα1-4)GlcNAcβ1-3Galβ1-4Glc	0.76	0.53	0.37	0.45	0.11	0.01
7E.	Lacto-N-difucohexose II	Galβ1-3(Fucα1-4)GlcNAcβ1-3Galβ1-4(Fucα1-3)Glc	2.18	0.18	0.32	0.14	0.33	0.36
7F.	H-disaccharide	Fucα1-2Gal	1.51	0.63	0.47	0.2	0.35	0.04
7G.	2'-Fucosyllactose	Fucα1-2Galβ1-4Glc	1.79	1.4	0.1	0.18	0.01	0.09
7H.	3'-Fucosyllactose	Galβ1-4(Fucα1-3)Glc	1.46	0.26	3.02	1.09	-0.36	0.2
7I.	Lewis^{x}	Galβ1-4(Fucα1-3)GlcNAc	2.78	1.21	1.81	0.6	1.82	1.66
7J.	Lewis^{a}	Galβ1-3(Fucα1-4)GlcNAc	1.39	0.34	0.26	0.05	0.28	0.01
7K.	Blood Group A-trisaccharide	GalNAcα1-3(Fucα1-2)Gal	1.07	0.37	2.29	0.05	1.88	0.16
7L.	Lactodifucotetrose	Fucα1-2Galβ1-4(Fucα1-3)Glc	1.31	1.36	6.49	2.15	0.11	0.15
7M.	Blood Group B-Trisaccharide	Galβ1-3(Fucα1-2)Gal	1.32	0.29	2.3	0.18	1.34	0.3
7N.	Lewis^{y}	Fucα1-2Galβ1-4(Fucα1-3)GlcNAc	2.66	1.05	0.35	0.27	0.13	0.34
7O.	Blood Group H Type II Trisaccharide	Fucα1-2Galβ1-3GlcNAc	1.79	0.52	1.35	0.45	1.37	1.22
7P.	Lewisb tetrasaccharide	Fucα1-2Galβ1-3(Fucα1-4)GlcNAc	1.32	0.32	0.39	0.04	0.39	0.01
8A.	Sulpho Lewisa	SO_3-3Galβ1-3(Fucα1-4)GlcNAc	1.11	0.15	0.15	0.07	0.11	0.04
8B.	Sulpho Lewisx	SO_3-3Galβ1-4(Fucα1-3)GlcNAc	1.28	0.12	0.27	0.03	0.27	0.19
8C.	Monofucosyl-para-Lacto-*N*-hexose IV	Galβ1-3GlcNAcβ1-3Galβ1-4(Fucα1-3)GlcNAcβ1-3Galβ1-4Glc	1.11	0.37	0.48	0.32	0.64	0.04
8D.	Monofucosyllacto-*N*-hexose III	Galβ1-4(Fucα1-3)GlcNAcβ1-6(Galβ1-3GlcNAcβ1-3)Galβ1-4Glc	-0.04	0.48	0.38	0.2	0.5	0.15
8E.	Difucosyllacto-*N*-hexose	Galβ1-4(Fucα1-3)GlcNAcβ1-6(Fucα1-2Galβ1-3GlcNAcβ1-3)Galβ1-4Glc	4.62	0.34	0.67	0	0.66	0.09
8F.	Trifucosyllacto-*N*-hexose	Galβ1-4(Fucα1-3)GlcNAcβ1-6(Fucα1-2Galβ1-3(Fucα1-4)GlcNAcβ1-3)Galβ1-4Glc	1.49	0.38	1.66	1.99	0.47	0.18
10A.	Sialyl Lewis^{a}	Neu5Acα2-3Galβ1-3(Fucα1-4)GlcNAc	3.83	2.11	1.3	0.18	1.13	0.16
10B.	Sialyl Lewis^{x}	Neu5Acα2-3Galβ1-4(Fucα1-3)GlcNAc	0.64	0.11	0.06	0.26	-0.11	0.13
10C.	Sialyllacto-*N*-tetrose a	Neu5Acα2-3Galβ1-3GlcNAcβ1-3Galβ1-4Glc	3.09	1.4	0.4	0.12	0.46	0.08

10D.	Monosialyl, monofucosyllacto-*N*-neohexose	Galβ1-4(Fucα1-3)GlcNAcβ1-6(Neu5Acα2-6Galβ1-4GlcNAcβ1-3)Galb1-4Glc	2.09	1	1.57	0.51	1.65	0.92
10E		Neu5Acα2-3Galβ1-3(Neu5Acα2-6)GalNAc	2.12	0.57	1.13	0.03	0.77	0.24
10K.	2,3'-Sialyllactosamine	Neu5Acα2-3Galβ1-4GlcNAc	3.23	0.75	0.7	0.46	0.87	0.35
10L.	2,6'-Sialyllactosamine	Neu5Acα2-6Galβ1-4GlcNAc	4.86	3.04	1.93	1.47	2.74	0.4
10M.	LS-Tetrasaccharide a		3.35	1.04	3.22	1.94	4	1.18
10N.	LS-Tetrasaccharide b	Galβ1-3(Neu5Acα2-6)GlcNAcβ1-3Galβ1-4Glc	6.09	2.69	1.74	1.18	2.3	0.25
10O.	LS-Tetrasaccharide c	Neu5Acα2-6Galβ1-4GlcNAcβ1-3Galβ1-4Glc	2.63	0.58	0.57	0.49	0.75	0.45
10P.	Disialyllacto-*N*-tetraose	Neu5Acα2-3Galβ1-3(Neu5Acα2-6)GlcNAcβ1-3Galβ1-4Glc	4.79	2.62	1.69	1.43	2.47	0.28
11A.	2,3'-Sialyllactose	Neu5Acα2-3Galβ1-4Glc	2.81	0.8	2.08	1.57	2.91	0.18
11B.	2,6'-Sialyllactose	Neu5Acα2-6Galβ1-4Glc	2.29	0.95	2.9	2.27	4.03	0.23
11C.	Colominic acid	(Neu5Acα2-8Neu5Ac)n (n<50)	3.47	1.85	1.92	1.27	2.48	0.53
11D.	Biantennary 2,6-sialylated-*N*-glycan-Asn	Neu5Acα2-6Galβ1-4GlcNAcβ1-2Manα1-6(Neu5Acα2-6Galβ1-4GlcNAcβ1-2Manα1-6)Manβ1-4GlcNAcβ1-4GlcNAc-Asn	2.32	0.82	0.41	0.17	0.31	0.07
12A.	Neocarratetrose-41, 3-di-*O*-sulphate (Na^+)	$C_{24}H_{36}O_{25}S_2Na_2$ (Mixed anomers. Tetrasaccharide of regular κ -carrageenan)	1.96	0.87	2.4	1.94	3.37	0.19
12B.	Neocarratetrose-41-*O*-sulphate (Na^+)	$C_{24}H_{37}O_{22}SNa$ (Mixed anomers. Derived from C1003 by removal of the non-reducing terminal 4-sulphate)	1.32	0.74	1.03	0.34	1.03	1
12C.	Neocarrahexose-24,41, 3, 5-tetra-*O*-sulphate (Na^+)	$C_{36}H_{52}O_{40}S_4Na_4$ (Mixed anomers. A hybrid sequence comprising carrageenan disaccharides in the order k-i-k, derived from the carrageenan from *Chondrus crispus*)	2.09	1.16	1.85	1.4	2.56	0.08
12D.	Neocarrahexose-41, 3, 5-tri-*O*-sulphate (Na^+)	$C_{36}H_{53}O_{37}S_3Na_3$ (Mixed anomers. Hexasaccharide of regular κ-carrageenan)	3.42	1.65	1.67	1.27	2.39	0.45
12E.	Neocarraoctose-41, 3, 5, 7-tetra-*O*-sulphate (Na^+)	$C_{48}H_{70}O_{49}S_4Na_4$ (Mixed anomers. Octasaccharide of regular κ-carrageenan)	1.81	1.65	1.88	1.42	2.69	0.56
12F.	Neocarradecose-41, 3, 5, 7, 9-penta-*O*-sulphate (Na^+)	$C_{60}H_{87}O_{61}S_5Na_5$ (Mixed anomers. Decasaccharide of regular κ-carrageenan)	1.74	0.55	0.42	0.02	0.4	0.13
12G.	ΔUA-2S GlcNS-6S Na_4 (I-S)	$C_{12}H_{15}NO_{19}S_3Na_4$ (Predominant disaccharide produced from heparin by heparinase I and II)	1.43	0.64	2.04	1.57	2.79	0.3
12H.	ΔUA-GlucNS-6S Na_3 (II-S)	$C_{12}H_{16}NO_{16}S_2Na_3$ (Produced from heparinase II digestion of heparin and heparin sulphate)	1.27	0.22	1.58	1.2	2.07	0.77
12I.	ΔUA-2S-GlcNS Na_3 (III-S)	$C_{12}H_{16}NO_{16}S_2Na_3$ (Produced from heparin by digestion with heparinase I and II)	4.77	4.02	1.89	1.11	2.54	0.66
12J.	ΔUA-2S-GlcNAc-6S Na_3 (I-A)	$C_{14}H_{18}NO_{17}S_2Na_3$ (Minor component produced from heparin by heparinase II)	3.06	0.81	1.14	0.05	1.1	0.2
12K.	ΔUA-GlcNAc-6S Na_2 (II-A)	$C_{14}H_{19}NO_{14}SNa_2$ (Product of the action of heparinases II and III on heparin and heparan sulphate)	2.26	1.32	2.21	1.33	2.84	0.22
12L.	ΔUA-2S-GlcNAc Na_2 (III-A)	$C_{14}H_{19}NO_{14}SNa_2$ (Minor product of the action of heparinase II on heparin)	3.54	1.18	1.63	1.09	2.12	0.4
12M.	ΔUA-GlcNAc Na (IV-A)	$C_{14}H_{20}NO_{11}Na$ (Produced from heparin sulphate by digestion With heparinase III)	1.39	0.5	2.18	1.59	3.02	0.23
12N.	ΔUA-GalNAc-4S Na_2 (Δ Di-4S)	$C_{14}H_{19}NO_{14}SNa_2$ (Produced from various chondroitin sulphates By the action of chondroitinases ABC, B and AC-1)	1.54	0.59	0.61	0.37	0.73	0.43
12O.	ΔUA-GalNAc-6S Na2 (Δ Di-6S)	C14H19NO14SNa2 (Produced from various chondroitin sulphates By the action of chondroitinases ABC, AC-1 and C)	3.62	2.3	2.28	1.8	3.09	0.77
12P.	ΔUA-GalNAc-4S,6S Na_3 (Δ Di-disE)	$C_{14}H_{18}NO_{17}S_2Na_3$ (Produced from various chondroitin sulphates By the action of chondroitinases ABC, B and AC-1)	5.37	5.55	2.08	1.35	2.64	0.77
13A.	ΔUA-2S-GalNAc-4S Na_2 (Δ Di-disB)	$C_{14}H_{18}NO_{17}S_2Na_3$ (Produced from various chondroitin sulphates by action of	9.67	3.3	2.15	0.96	0.84	0.2

		chondroitinase ABC and/or B. Most typically from chondroitin sulphate B (dermatan sulphate))						
13B.	ΔUA -2S-GalNAc-6S Na_3 (Δ Di-disD)	$C_{14}H_{18}NO_{17}S_2Na_3$ (Produced from various chondroitin sulphates by the action of chondroitinase ABC)	2.23	1.33	1.21	0.4	1.25	0.95
13C.	ΔUA-2S-GalNAc-4S-6S Na_4 (Δ Di-tisS)	$C_{14}H_{17}NO_{20}S_3Na_4$ (Produced as a minor component by the action of chondroitinase ABC on various chondroitin sulphates, particularly B)	3.54	1.55	1.93	0.21	1.63	0.74
13D.	ΔUA-2S-GalNAc-6S Na_2 (ΔDi-UA2S)	$C_{14}H_{19}NO_{14}SNa_2$ (Produced as a minor component from various chondroitin sulphates by the action of chondroitinase ABC)	1.47	0.39	2.06	0.66	2.11	1.25
13E.	ΔUA-GlcNAc Na (Δ Di-HA)	$C_{14}H_{20}NO_{11}Na$ (The only unsaturated disaccharide produced from hyaluronic acid by the action of chondroitinase ABC or AC-1)	2.25	0.94	2.38	1.97	3.19	1.35
13F.	Hyaluronan fragments (4mer)	(GlcAβ1-3GlcNAcβ1-4)n (n=4)	2.55	1.25	0.74	0.44	0.87	0.59
13G.	Hyaluronan fragments (8mer)	(GlcAβ1-3GlcNAcβ1-4)n (n=8)	5.62	2.57	0.59	0.22	0.67	0.15
13H.	Hyaluronan fragments (10mer)	(GlcAβ1-3GlcNAcβ1-4)n (n=10)	2.4	0.63	1.43	0.08	1.23	0.69
13I.	Hyaluronan fragments (12mer)	(GlcAβ1-3GlcNAcβ1-4)n (n=12)	2.22	1.17	0.61	0.02	0.57	0.07
13J.	Heparin	(GlcA/IdoAα/β1-4GlcNAcα1-4)n (n=200)	1.5	0.79	1.88	1.28	2.43	0.58
13K.	Chondroitin sulfate	(GlcA/IdoAβ1-3(±4/6S)GalNAcβ1-4)n (n<250)	2.1	1.18	2.11	1.71	2.96	0.21
13L.	Dermatan sulfate	((±2S)GlcA/IdoAα/b1-3(±4S)GalNAcβ1-4)n (n<250)	1.62	0.61	1.88	1.21	2.56	0.49
13M.	Chondroitin 6-Sulfate	(GlcA/IdoAβ1-3(±6S)GalNAcβ1-4)n (n<250)	2.12	0.07	1.45	0.98	1.75	1.27
13N.	HA-4 (10mM)	(GlcAβ1-3GlcNAcβ1-4)n (n=4)	3.31	1.59	2.35	0.98	2.74	0.44
13O.	HA-6 (10mM)	(GlcAβ1-3GlcNAcβ1-4)n (n=6)	5.93	5.11	2.01	0.62	2.19	0.61
13P.	HA-8 (9.7mM)	(GlcAβ1-3GlcNAcβ1-4)n (n=8)	2.66	1.27	0.54	0.24	0.64	0.08
14A.	HA-10 (7.83mM)	(GlcAβ1-3GlcNAcβ1-4)n (n=10)	4.26	1.89	2.11	0.7	0.41	0.44
14B.	HA-12 (6.5mM)	(GlcAβ1-3GlcNAcβ1-4)n (n=12)	3.99	0.91	1.24	0	1.08	0.74
14C.	HA-14 (5.6mM)	(GlcAβ1-3GlcNAcβ1-4)n (n=14)	3.27	0.74	0.64	0.39	0.77	0.46
14D.	HA-16 (4.9mM)	(GlcAβ1-3GlcNAcβ1-4)n (n=16)	3.73	0.79	2.05	1.63	2.92	0.21
14E.	HA-30,000da 2.5mg/ml	(GlcAβ1-3Glc*N*Ac β1-4)n	1.54	0.63	1.73	1.31	2.37	0.22
14F.	HA-107,000da 2.5mg/ml	(GlcAβ1-3Glc*N*Ac β1-4)n	0.82	0.1	1.78	1.28	2.37	0.46
14G.	HA-190,000da 2.5mg/ml	(GlcAβ1-3Glc*N*Ac β1-4)n	1.78	1.3	2.86	2.13	3.92	0.21
14H.	HA-220,000da 2.5mg/ml	(GlcAβ1-3Glc*N*Ac β1-4)n	0.63	0.56	1.45	1.01	1.74	1.41
14I.	HA-1,600,000da 2.5mg/ml	(GlcAβ1-3Glc*N*Ac β1-4)n	1.02	0.74	0.49	0.04	0.48	0.13
14J.	Heparin Sulfate 5mg/ml	(GlcA/IdoAα/ IdoASα/β1-4Glc*N*Ac/GlcNS/Glc*N*Ac6Sα1-4)n	1.98	1.39	0.52	0.32	0.64	0.29
14K.	Beta-1-3Glucan	(Glcβ1-3Glcβ1-3)n	1.12	0.32	0.33	0.26	0.44	0.14
1	L-α-Fuc	Fucα-sp3	0.27	0.14	0.5	0.15	0.55	0.14
2	α-Gal	Galα-sp3	1.6	1.08	0.33	0.04	0.29	0.32
3	β-Gal	Galβ-sp3	0.89	0.2	0.36	0.15	0.42	0.04
4	TnSer	GalNAcα-sp0	0.29	0.13	0.71	0.31	0.79	0.46
5	T_n	GalNAcα-sp3	0.72	0.13	2.96	0.98	2.97	2.73
6	β-GalNAc	GalNAcβ-sp3	0.54	0.22	0.06	0.18	-0.03	0.09
7	α-Glc	Glcα-sp3	0.55	0.27	0.14	0.04	0.07	0.57
9	β-Glc	Glcβ-sp3	0.54	0.19	0.43	0.04	0.38	0.37
10	β-GlcNAc	GlcNAcβ-sp3	0.79	0.26	0.38	0.17	0.45	0.06
14	β-GlcN(Gc)	GlcN(Gc)β-sp4	0.65	0.13	0.47	0.17	0.55	0.06

15	aminoglucitol	$HOCH_2(HOCH)_4CH_2NH_2$	0.39	0.12	0.47	0.02	0.43	0.2
16	α-Man	Manα-sp3	1.45	0.3	0.55	0.44	0.75	0.16
18	β-Man	Manβ-sp4	1.25	0.9	0.55	0.19	0.56	0.49
19	β-ManAc	ManNAcβ-sp4	2.37	1.09	0.11	0.11	0.07	0.14
20	L-α-Rha	Rhaα-sp3	1.5	0.39	0.42	0.17	0.48	0.11
22	β-GlcNAc	GlcNAcβ-sp4	0.08	0.32	0.63	0.29	0.5	0.39
37	3-O-Su-β-Gal	3-O-Su-Galβ-sp3	0.65	0.3	0.43	0.02	0.47	0.41
38	3-O-Su-β-GalNac	3-O-Su-GalNAcα-sp3	0.47	0.26	0.24	0.08	0.24	0.25
43	6-O-Su-β-GlcNAc	6-O-Su-GlcNAcβ-sp3	0.19	0.34	0.66	0.34	0.89	0.41
44	α-glucoronic acid	GlcAα-sp3	0.42	0.45	0.23	0.21	0.33	0.06
45	β-glucoronic acid	GlcAβ-sp3	0.41	0.55	0.52	0.19	0.57	0.19
46	β-Glc6P	$6\text{-}H_2PO_3$Glcβ-sp4	0.85	0.2	0.42	0.4	0.59	0.29
47	α-Man6P	$6\text{-}H_2PO_3$Manα-sp3	0.6	0.17	0.03	0.13	-0.05	0.02
48	α-Neu5Ac	Neu5Acα-sp3	0.41	0.24	0.34	0.3	0.45	0.29
49	α-Neu5AcBn	Neu5Acα-sp9	0.12	0.14	0.3	0.1	0.35	0.05
52	α-Neu5Gc	Neu5Gcα-sp3	1.13	0.78	0.09	0.02	0.09	0.07
54	9-Nac-α-Neu5Ac	9-NAc-Neu5Acα-sp3	0.61	0.13	0.51	0.1	0.5	0.32
55	3-O-Su-β-GlcNAc	3-O-Su-GlcNAcβ-sp3	1.05	0.5	1.03	0.34	1.06	0.77
71	H_{di}	Fucα1-2Galβ-sp3	1.07	0.59	0.51	0.04	0.46	0.1
72		Fucα1-3GlcNAcβ-sp3	0.76	0.21	0.38	0.13	0.43	0.02
73	Le	Fucα1-4GlcNAcβ-sp3	0.69	0.83	0.11	0.05	0.06	0.16
75		Galα1-2Galβ-sp3	0.56	0.32	0.08	0.08	0.1	0.15
76	B_{di}	Galα1-3Galβ-sp3	1.06	0.53	0.59	0.39	0.38	0.17
77	$T_{\alpha\beta}$	Galα1-3GalNAcβ-sp3	0.86	0.59	0.52	0.05	0.37	0.65
78	$T_{\alpha\alpha}$	Galα1-3GalNAcα-sp3	1.46	0.82	0.78	0.07	0.64	0.42
80		Galα1-3GlcNAcβ-sp3	0.52	0.1	0.28	0.01	0.24	0.2
81	α-LacNAc	Galα1-4GlcNAcβ-sp3	1.95	0.48	1.82	0.31	0.18	0.25
83	Melibiose	Galα1-6Glcβ-sp4	1.64	1	0.32	0.15	0.37	0.1
84		Galβ1-2Galβ-sp3	1.74	0.33	0.54	0.08	0.56	0.04
85	Le^c	Galβ1-3GlcNaAcβ-sp3	3.99	1.24	0.11	0.34	-0.07	0.07
87		Galβ1-3Galβ-sp3	0.72	0.39	0.14	0.19	0.01	0.15
88	$T_{\beta\beta}$	Galβ1-3GalNAcβ-sp3	0.88	0.4	0.59	0.2	0.68	0.04
89	TF	Galβ1-3GalNAcα-sp3	0.69	0.65	0.4	0.15	0.48	0.05
93	Lac	Galβ1-4Glcβ-sp4	4.04	0.73	0.23	0.5	-0.02	0.27
94		Galβ1-4Galβ-sp4	3.3	1.01	0.23	0.23	0.17	0.47
97	LacNAc	Galβ1-4GlcNAcβ-sp3	2.18	1.24	0.6	0.25	0.69	0.22
100		Galβ1-6Galβ-sp4	1.35	0.29	0.15	0.03	0.11	0.31
101	Fs-2	GalNAcα1-3GalNAcβ-sp3	1.02	0.76	0.18	0.15	0.27	0.11
102	A_{di}	GalNAcα1-3Galβ-sp3	1.49	1.23	0.11	0.13	0	0.24
103	core 5	GalNAcα1-3GalNAcα-sp3	2.43	1.15	0.38	0.16	0.52	0.45
104		GalNAcβ1-3Galβ-sp3	0.59	0.26	0.49	0.27	0.57	0.32
106	LacdiNAc	GalNAcβ1-4GlcNAcβ-sp3	1.46	1.63	0.54	0.2	0.7	0.45
110	maltose	Glcα1-4Glcβ-sp3	1.48	0.57	0.54	0.02	0.47	0.24
111	cellobiose	Glcβ1-4Glcβ-sp4	0.37	0.04	1.69	0.97	1.9	1.71
112	gentiobiose	Glcβ1-6Glcβ-sp4	0.72	0.3	0.16	0.08	0.22	0.13
113	core 3	GlcNAcβ1-3GalNAcα-sp3	1.16	0.99	0.45	0.12	0.5	0.02

114		GlcNAcβ1-3Manβ-sp4	2.84	0.31	0.42	0.02	0.44	0.09
115	chitobiose-Asn	GlcNAcβ1-4GlcNAcβ-Asn	1.7	0.86	0.65	0.46	0.89	0.06
117	chitobiose	GlcNAcβ1-4GlcNAcβ-sp4	0.59	0.98	0.25	0.03	0.23	0.14
118	core 6	GlcNAcβ1-6GalNAcα-sp3	1.01	0.67	1.19	0.03	1.27	0.85
119		Manα1-2Manβ-sp4	0.52	0.34	1.44	0.97	2.07	0.92
120		Manα1-3Manβ-sp4	1.37	0.21	1.38	1.31	2.15	0.6
121		Manα1-4Manβ-sp4	3.23	3.19	1.53	1.24	2.22	0.37
122		Manα1-6Manβ-sp4	-0.14	1.58	1.7	1.23	2.33	0.08
123		Manβ1-4GlcNAcβ-sp4	0.15	0.19	1.05	0.86	1.64	0.96
124		Manα1-2Manα-sp4	3.41	3.82	0.29	0.03	0.28	0.13
145	6-O-Su-Le^{c}	Galβ1-3(6-O-Su)GlcNAcβ-sp3	1.61	1.07	0.63	0.46	0.95	0.6
146	6-O-Su-Lac	Galβ1-4(6-O-Su)Glcβ-sp2	0.9	0.02	0.84	0.13	0.8	0.39
147	6-O-Su-LacNAc	Galβ1-4(6-O-Su)GlcNAcβ-sp3	0.7	1.43	1.48	1.09	2.17	0.83
149	6-O-Su-chitobiose	GlcNAcβ1-4(6-O-Su)GlcNAcβ-sp2	1.45	0.51	2.03	1.5	2.92	0.82
150	3`-O-Su-TF	3-O-Su-Galβ1-3GalNAcα-sp3	0.78	0.11	2.27	1.18	2.82	0.21
151	6`-O-Su-TF	6-O-Su-Galβ1-3GalNAcα-sp3	2.61	2.33	1.14	0.38	1.42	0.77
152	SM3	3-O-Su-Galβ1-4Glcβ-sp2	1.59	0.76	1.27	0.78	1.69	0.19
153	6`-O-Su-Lac	6-O-Su-Galβ1-4Glcβ-sp2	2.69	2.77	1.68	1.19	2.07	1.37
155	3`-O-Su-Le^{c}	3-O-Su-Galβ1-3GlcNAcβ-sp3	1.07	0.64	0.18	0.15	0.12	0.17
157	3`-O-Su-LacNAc	3-O-Su-Galβ1-4GlcNAcβ-sp3	-1.2	3.14	2.13	1.05	2.67	0.24
159	4`-O-Su-LacNAc	4-O-Su-Galβ1-4GlcNAcβ-sp3	0.27	1.03	0.57	0.56	0.25	0.07
161	6`-O-Su-Le^{c}	6-O-Su-Galβ1-3GlcNAcβ-sp3	1.52	1.37	2.12	1.65	2.95	0.13
163	6`-O-Su-LacNAc	6-O-Su-Galβ1-4GlcNAcβ-sp3	0.98	0.75	1.22	0.6	1.73	1.37
164		GlcAβ1-3GlcNAcβ-sp3	-0.32	0.07	1.94	1.47	2.63	0.38
165		GlcAβ1-3Galβ-sp3	0.39	0.51	2.05	1.49	2.8	0.12
166		GlcAβ1-6Galβ-sp3	2.7	1.84	1.86	1.6	2.71	0.16
167	GlcNAc-Mur	GlcNAcβ1-4-[HOOC(CH_3)CH]-3-O-GlcNAcβ-sp4	-0.44	1.52	1.02	0.69	1.29	0.46
168	GMDP-Lys	GlcNAcβ1--[HOOC(CH_3)CH]-3-O-GlcNAcβ-L-alanyl-D-i-glutaminyl-L-lysine	-1.07	2.17	2.21	1.65	2.99	0.39
169	GM4	Neu5Acα2-3Galβ-sp3	1.71	1.27	1.39	0.57	1.84	1.17
170		Neu5Acα2-6Galβ-sp3	0.15	0.51	1.37	0.99	1.96	0.52
171	3-SiaT$_{n}$	Neu5Acα2-3GalNAcα-sp3	-0.03	0.31	0.22	0.07	0.17	0.03
172	SiaT$_{n}$	Neu5Acα2-6GalNAcα-sp3	0.27	0.56	1.11	0.81	1.61	0.57
174	NeuGc-T$_{n}$	Neu5Gcα2-6GalNAcα-sp3	-0.44	0.57	1.76	1.47	2.45	0.4
176	3`,6-di-O-Su-Lac	3-O-Su-Galβ1-4(6-O-Su)Glcβ-sp2	1.97	0.71	1.96	1.48	2.65	0.46
177	3`,6-di-O-Su-LacNAc	3-O-Su-Galβ1-4(6-O-Su)GlcNAcβ-sp2	0.27	0.93	1.86	1.19	2.48	0.18
178	6,6`-di-O-Su-Lac	6-O-Su-Galβ1-4(6-O-Su)Glcβ-sp2	1.46	1.56	1.83	0.99	2.36	0.31
179	6,6`-di-O-Su-Le^{c}	6-O-Su-Galβ1-3(6-O-Su)GlcNAcβ-sp2	1.14	0.35	1.37	0.59	1.82	1.1
180	6,6`-di-O-Su-LacNAc	6-O-Su-Galβ1-4(6-O-Su)GlcNAcβ-sp2	0.77	0.69	1.86	1.4	2.62	0.27
181	3`,4`-di-O-Su-LacNAc	3,4-O-Su$_2$-Galβ1-4GlcNAcβ-sp3	0.12	0.14	1.5	1.13	2.14	0.38
182	3`,6`-di-O-Su-LacNAc	3,6-O-Su$_2$-Galβ1-4GlcNAcβ-sp2	-0.41	1.93	2.08	1.56	2.87	0.09
183	4`,6`-di-O-Su-LacNAc	4,6-O-Su$_2$-Galβ1-4GlcNAcβ-sp2	0.2	0.43	3.55	2.67	4.66	1.62
184	4`,6`-di-O-Su-LacNAc	4,6-O-Su$_2$-Galβ1-4GlcNAcβ-sp3	2.34	1.52	1.88	1.48	2.54	0.68
186	(Sia)$_2$	Neu5Acα2-8Neu5Acα2-sp3	1.9	2.25	2.16	1.65	2.99	0.11
189	3`,6,6`-tri-O-Su-	3,6-O-Su$_2$-Galβ1-4(6-O-Su)GlcNAcβ-sp2	1.23	0.47	1.39	1.04	1.99	0.49

	LacNAc							
192	6-O-Su-LacdiNAc	GalNAcβ1-4(6-O-Su)GlcNAcβ-sp3	0.84	0.13	2.31	1.51	3.12	0.36
193	3`-O-Su-LacdiNAc	3-O-Su-GalNAcβ1-4GlcNAcβ-sp3	2.01	1.64	1.43	0.98	1.94	0.12
194	6`-O-Su-LacdiNAc	6-O-Su-GalNAcβ1-4GlcNAcβ-sp3	0.46	0.32	0.9	0.84	1.37	0.2
195	6`-Su-3-O-Ac-LacdiNAc	6-O-Su-GalNAcβ1-4-(3-O-Su)GlcNAcβ-sp3	-0.67	1.96	2.57	2.02	3.45	1.03
196	3,3`-O-Su$_2$-LacdiNAc	3-O-Su-GalNAcβ1-4(3-O-Su)-GlcNAcβ-sp3	0.78	0.98	0.71	0.43	0.9	0.12
197	3`,6`-Su$_2$-LacdiNAc	3,6-O-Su$_2$-GalNAcβ1-4GlcNAcβ-sp3	3.16	2.94	2.17	1.55	2.85	0.68
198	4`,6`-Su$_2$-LacdiNAc	4,6-O-Su$_2$-GalNAcβ1-4GlcNAcβ-sp3	2.11	0.45	1.73	1.35	2.35	0.45
199	4`,6`-Su$_2$-3-O-Ac-LacdiNAc	4,6-O-Su$_2$-GalNAcβ1-4-(3-O-Ac)GlcNAcβ-sp3	1.36	0.75	0.91	0.2	1.1	0.76
200	4`-O-Su-LacdiNAc	4-O-Su-GalNAcβ1-4GlcNAcβ-sp3	0.61	0.41	1.82	1.33	2.68	1.23
201	3`,4`-Su$_2$-LacdiNAc	3,4-O-Su$_2$-Galβ1-4GlcNAcβ-sp3	3.75	4.6	1.6	1.14	2.24	0.38
202	6,6`-O-Su-LacdiNAc	6-O-Su-GalNAcβ1-4(6-O-Su)GlcNAcβ-sp3	0.81	0.2	0.45	0.3	0.64	0.25
203	6-O-Su-LacNAc	Galβ1-4(6-O-Su)GlcNAcβ-sp2	2.62	1.89	1.7	1.14	2.26	0.11
204	4`-O-Su-LacdiNAc	4-O-Su-GalNAcβ1-4GlcNAcβ-sp2	0.8	0.64	0.69	0.47	0.96	0.2
205		Neu5Acα2-6GalNAcβ-sp3	-4.15	7.76	0.6	0.4	0.83	0.12
206	NeuGcα3Gal	Neu5Gcα2-3Gal-sp3	0.61	0.68	2.21	1.76	3.08	0.23
215	Le^{d}, H (type 1)	Fucα1-2Galβ1-3GlcNAcβ-sp3	1.36	0.28	1.17	0.99	0.58	0.03
216	H (type 2)	Fucα1-2Galβ1-4GlcNAcβ-sp3	1.97	0.62	2.07	1.76	2.99	0.1
217	H (type 3)	Fucα1-2Galβ1-3GalNAcα-sp3	-0.02	0.78	0.61	0.28	0.49	0.35
219	H (type 6)	Fucα1-2Galβ1-4Glcβ-sp4	3.08	2.94	0.41	0.01	0.61	0.71
220		Galα1-3Galβ1-4Glcβ-sp2	0.17	0.59	1.15	0.6	1.27	1.14
222	Galili (tri)	Galα1-3Galβ1-4GlcNAcβ-sp3	0.6	1.11	0.18	0.08	0.23	0.05
224	P^{k}, Gb3, GbOse$_3$	Galα1-4Galβ1-4Glcβ-sp3	2.37	4.03	1.88	1.63	2.54	1.17
225	P$_1$	Galα1-4Galβ1-4GlcNAc-sp2	0.33	0.17	2.01	1.43	2.89	0.99
226	B$_{tri}$	Galα1-3(Fucα1-2)Galβ-sp3	1.34	0.43	1.99	1.4	2.75	0.32
228		Galβ1-2Galα1-4GlcNAcβ-sp4	3.13	1.65	2.09	1.61	2.97	0.36
229		Galβ1-3Galβ1-4GlcNAcβ-sp4	1.88	1.3	2	1.69	2.92	0.34
231		Galβ1-4GlcNAcβ1-3GalNAcα-sp3	0.51	0.22	2.16	1.17	2.79	1.08
232		Galβ1-4GlcNAcβ1-6GalNAcα-sp3	0.59	0.34	2.4	1.77	3.36	0.4
233	Le^{a}	Galβ1-3(Fucα1-4)GlcNAcβ-sp3	3.19	1.88	2.93	2.21	4.05	0.15
234	Le^{x}	Galβ1-4(Fucα1-3)GlcNAcβ-sp3	1.34	0.49	4.19	3.06	6.15	2.58
235	A$_{tri}$	GalNAcα1-3(Fucα1-2)Galβ-sp3	1.28	0.07	2.07	1.29	2.75	0.29
238	GA$_2$, GgOse$_3$	GalNAcβ1-4Galβ1-4Glcβ-sp3	2.46	0.7	1.97	1.52	2.74	0.1
240	maltotriose	(Glcα1-4)$_3$β-sp4	0.53	0.36	1.3	0.77	1.77	0.56
241	isomaltotriose	(Glcα1-6)$_3$β-sp4	0.67	0.24	1.66	1.39	2.5	0.77
246		GlcNAcβ1-2Galβ1-3GalNAcα-sp3	1.15	0.17	2.47	2.11	3.54	0.16
247		GlcNAcβ1-3Galβ1-3GalNAcα-sp3	1.34	0.16	2.16	1.8	3.14	0.31
248		GlcNAcβ1-3Galβ1-4Glcβ-sp2	0.77	0.31	2.12	1.73	3.11	0.64
250		GlcNAcβ1-3Galβ1-4GlcNAcβ-sp3	0.83	0.12	2.77	2.26	4.02	0.58
251		GlcNAcβ1-4Galβ1-4GlcNAcβ-sp2	0.89	1.01	2.44	1.62	3.29	0.26
252	chitotriose	GlcNAcβ1-4GlcNAcβ1-4GlcNAcβ-sp4	0.36	0.63	1.94	1.51	2.84	0.78
253		GlcNAcβ1-6Galβ1-4GlcNAcβ-sp2	0.96	0.53	2.39	1.78	3.46	1.03
254	core 2	GlcNAcβ1-6(Galβ1-3)GalNAcα-sp3	1.09	0.25	2.55	1.91	3.55	0.17
255	core 4	GlcNAcβ1-6(GlcNAcβ1-3)GalNAcα-sp3	1.31	0.38	2.1	1.28	2.74	0.09
258	Man$_3$	Manα1-6 (Manα1-3)Manβ-sp4	1.37	0.5	2.48	2.59	3.94	0.7

262	$T_{\beta\beta}$-Gal	Galβ1-3GalNAcβ1-3Gal-sp4	2.03	0.73	2.09	1.59	3.01	0.68
264		Galβ1-4Galβ1-4GlcNAc-sp3	2.95	0.9	2.05	1.05	2.58	0.12
287	Su-Le^{a}	Fucα1-4(3-O-Su-Galβ1-3)GlcNAcβ-sp3	0.54	0.31	1.5	0.85	2.09	1.09
288	Su-Le^{x}	3-O-Su-Galβ1-4(Fucα1-3)GlcNAcβ-sp3	1.27	0.57	2.17	1.88	3.17	0.21
289	6-SiaTF	Neu5Acα2-6(Galα1-3)GalNAcα-sp3	0.02	0.49	1.85	1.3	2.56	0.32
290		Neu5Acα2-6(Galβ1-3)GalNAcα-sp3	0.47	0.49	0.04	0.08	0.02	0.04
292	3`-Sia-TF	Neu5Acα2-3Galβ1-3GalNAcα-sp3	0.64	1.24	2.53	1.71	3.37	0.2
293	3`SL	Neu5Acα2-3Galβ1-4Glcβ-sp3	2.49	1.33	2.43	2.18	3.62	0.4
294	3`SL	Neu5Acα2-3Galβ1-4Glcβ-sp4	0.64	0.08	3.18	2.64	4.51	0.2
295	6`SL	Neu5Acα2-6Galβ1-4Glcβ-sp2	2.29	0.94	2.09	0.9	2.51	0.08
298	3`SLN	Neu5Acα2-3Galβ1-4GlcNAcβ-sp3	0	0.2	1.67	1.17	2.26	0.07
299	3`-SiaLe^{c}	Neu5Acα2-3Galβ1-3GlcNAcβ-sp3	0.43	0.53	1.89	1.54	2.74	0.42
300	6`SLN	Neu5Acα2-6Galβ1-4GlcNAcβ-sp3	1.11	0.41	2.19	1.8	3.12	0.11
303	3`SLN (Gc)	Neu5Gcα2-3Galβ1-4GlcNAcβ-sp3	0.88	0.56	1.85	1.42	2.6	0.17
304	6`SLN (Gc)	Neu5Gcα2-6Galβ1-4GlcNAcβ-sp3	1.33	0.62	2.02	1.25	2.71	0.46
306		9-NAc-Neu5Acα2-6Galβ1-4GlcNAcβ-sp3	0.39	0.14	3.98	3.17	6.1	3.22
315	6-Su-3`SLN	Neu5Acα2-3Galβ1-4-(6-O-Su)GlcNAcβ-sp3	0.77	0.04	2.79	2.27	4.02	0.41
317	6-Su-3`SiaTF	Neu5Acα2-3Galβ1-3-(6-O-Su)GalNAcβ-sp3	1.03	0.12	2.49	1.22	3.19	0.76
318	6-Su-6`SLN	Neu5Acα2-6Galβ1-4-(6-O-Su)GlcNAcβ-sp3	0.23	0.44	2.07	1.64	3.04	0.88
319	6`-Su-3`SLN	Neu5Acα2-3-(6-O-Su)Galβ1-4GlcNAcβ-sp3	0.97	0.1	2.55	1.89	3.46	0.37
321	$(Sia)_{3}$	$(Neu5Ac\alpha2\text{-}8)_{3}$-sp3	1.02	0.3	3.16	2.59	4.45	0.31
323	6`-SiaLe^{c}	Neu5Acα2-6Galβ1-3GlcNAc-sp3	0.64	0.62	0.6	0.31	0.82	0.48
324	6Su-6`-SiaLe^{c}	Neu5Acα2-6Galβ1-3(6-O-Su)GlcNAc-sp3	1.71	0.72	1.16	0.53	1.34	0.51
331	3`SiaLe^{c} (Gc)	Neu5Gcα2-3Galβ1-3GlcNAcβ-sp3	0.71	0.19	1.65	0.29	1.86	0.69
359	B (type 1)	Galα1-3(Fucα1-2)Galβ1-3GlcNAcβ-sp3	0.02	1.03	4.42	3.46	5.7	3.21
360	B (type 2)	Galα1-3(Fucα1-2)Galβ1-4GlcNAcβ-sp3	4.32	1.82	3.51	2.67	4.82	0.41
362	B (type 3)	Galα1-3(Fucα1-2)Galβ1-3GalNAcα-sp3	0.71	0.14	1.96	1.24	2.69	0.7
363	B (type 4)	Galα1-3(Fucα1-2)Galβ1-3GalNAcβ-sp3	2.52	2.02	1.84	1.37	2.07	0.57
364	αGalLe^{x}	Galα1-3Galβ1-4(Fucα1-3)GlcNAcβ-sp3	2.68	1.42	1.75	1.42	2.58	0.71
366	A (type 1)	GalNAcα1-3(Fucα1-2)Galβ1-3GlcNAcβ-sp3	1.5	0.03	1.8	0.26	1.65	0.18
368	A (type 2)	GalNAcα1-3(Fucα1-2)Galβ1-4GlcNAcβ-sp3	1.24	0.77	5.94	4.31	7.32	5.24
371	Le^{b}	Fucα1-4(Fucα1-2Galβ1-3)GlcNAcβ-sp3	1.32	0.36	1.61	1.28	2.38	0.75
372	Le^{y}	Fucα1-2Galβ1-4(Fucα1-3)GlcNAcβ-sp3	1.12	0.71	1.99	1.13	2.67	0.77
373	Galili (tetra)	Galα1-3Galβ1-4GlcNAcβ1-3Galβ-sp3	0.91	0.98	3.68	1.81	4.7	0.95
375		Galα1-4GlcNAcβ1-3Galβ1-4GlcNAcβ-sp3	5.94	1.92	3.55	2.92	5.49	2.87
376	LNT	Galβ1-3GlcNAcβ1-3Galβ1-4Glcβ-sp4	1.58	0.63	1.28	1.02	0.51	0.69
377		Galβ1-3GlcNAcβ1-3Galβ1-3GlcNAcβ-sp2	0.36	0.58	2.43	2	3.68	1.42
378		Galβ1-3GlcNAcα1-3Galβ1-4GlcNAcβ-sp3	0.53	0.26	2.74	2.09	3.86	0.33
379		Galβ1-3GlcNAcβ1-3Galβ1-4GlcNAcβ-sp3	1.34	0.56	2.9	2.3	4.26	1.19
380		Galβ1-3GlcNAcα1-6Galβ1-4GlcNAcβ-sp2	1.97	0.57	2.55	0.74	3.01	0.89
381		Galβ1-3GlcNAcβ1-6Galβ1-4GlcNAcβ-sp2	0.8	0.21	2	0.77	2.56	1.33
382	Asialo-GM1	Galβ1-3GalNAcβ1-4Galβ1-4Glcβ-sp3	2.24	1.07	0.25	0.1	0.34	0.29
383	LNnT	Galβ1-4GlcNAcβ1-3Galβ1-4Glcβ-sp2	3.67	3.03	1.56	1.02	2	0.51

385	i	Galβ1-4GlcNAcβ1-3Galβ1-4GlcNAcβ-sp3	5.37	1.35	1.68	1.03	2.17	0.17
387		Galβ1-4GlcNAcβ1-6Galβ1-4GlcNAcβ-sp2	1.55	1.28	1.41	1.07	1.94	0.14
388		Galβ1-4GlcNAcβ1-6(Galβ1-3)GalNAcα-sp3	3.67	1.11	1.16	0.38	1.21	0.78
389	Gb4, P	GalNacβ1-3GalαGalβ1-4Glcβ-sp3	0.16	0.13	0.17	0.1	0.2	0.14
390	maltotetraose	(Glcα1-4)$_4$β-sp4	5.25	1.29	0.21	0.01	0.18	0.07
391	isomaltotetraose	(Glcα1-6)$_4$β-sp4	3.54	0.73	0.15	0.3	0.3	0.11
392	A (type 3)	GalNAcα1-6(Fucα1-2)Galβ1-3GalNAcα-sp3	1.34	0.26	2.29	0.07	1.26	0.15
395	Tk	GlcNAcβ1-6(GlcNAcβ1-3)Galβ1-4GlcNAcβ-sp3	5.5	3.84	1.13	0.38	1.3	0.04
401	Le^{c}3Le^c	Galβ1-3GlcNAcβ1-3Galβ1-3GlcNAcβ-sp3	0.97	0.2	1.34	0.99	1.78	0.39
419		3-O-SuGalβ1-4GlcNAcβ1-3Galβ1-4GlcNAcβ-sp3	0.41	0.26	2.33	1.69	3.06	0.88
420		4-O-SuGalβ1-4GlcNAcβ1-3Galβ1-4GlcNAcβ-sp3	1.77	0.63	1.01	0.33	1.1	0.42
421	GM2	GalNAcβ1-4(Neu5Acα2-3)Galβ1-4Glcβ-sp2	1.2	0.88	0.45	0.05	0.43	0.17
422		Neu5Acα2-3Galβ1-4GlcNAcβ1-3Galβ-sp3	4.86	2.65	0.26	0.06	0.18	0.22
423	SiaLe^x	Neu5Acα2-3Galβ1-4(Fucα1-3)GlcNAcβ-sp3	1.02	1.35	0.24	0.07	0.27	0.02
426	SiaLe^a	Neu5Acα2-3(Fucα1-4)GlcNAcβ-sp3	2.44	2.46	2.06	1.62	2.75	0.88
428		Neu5Acα2-3Galβ1-4(Fucα1-3)6-O-Su-GlcNAcβ-sp3	1.37	1.23	2.19	1.54	2.75	1.38
429		Neu5Acα2-3(6-O-Su)Galβ1-4(Fucα1-3)GlcNAcβ-sp3	0.61	0.28	1.92	1.49	2.61	0.42
433	Sia$_2$-TF	Neu5Acα2-6(Neu5Acα2-3Galβ1-3)GalNAcα-sp3	2.2	1.25	1.46	0.64	1.77	0.08
434	GD3	Neu5Acα2-8Neu5Acα2-3Galβ1-4Glcβ-sp4	1.82	0.59	3.84	2.01	4.52	1.94
479	LNFP-I	Fucα1-2Galβ1-3GlcNAcβ1-3Galβ1-4Glcβ-sp4	1.11	0.16	5.45	4.59	7.01	5.14
480	H (type1) penta	Fucα1-2Galβ1-3GlcNAcβ1-3Galβ1-4GlcNAcβ-sp2	1.31	2.03	2.05	0.07	0.09	0.02
481	Galili (penta)	Galα1-3Galβ1-4GlcNAcβ1-3Galβ1-4Glcβ-sp4	1.29	0.67	0.49	0.03	0.43	0.16
483	Ble^y	Galα1-3(Galβ1-4)Fucα1-2(Fucα1-3)GlcNAcβ-sp3	-0.24	0.7	0.59	0.6	0.21	0.17
488		Galβ1-4GlcNAcβ1-6(Galβ1-4GlcNAcβ1-3)GalNAcα-sp3	0.68	0.63	0.45	0.1	0.59	0.67
489		Galβ1-4GlcNAcβ1-3(GlcNAcβ1-6)Galβ1-4GlcNAc-sp2	0.72	0.87	3.76	3.06	5.13	1.31
490		Galβ1-4GlcNAcβ1-6(GlcNAcβ1-3)Galβ1-4GlcNAcβ-sp2	0.19	0.4	1.4	0.28	1.53	0.14
492	isomaltopentaose	(Glcα1-6)$_5$β-sp4	4.74	1.33	1.94	1.45	2.58	0.66
493	chitopentaose	(GlcNAcβ1-4)$_5$β-sp4	4.03	3.46	0.58	0.68	0.19	0.06
495	Man5	Manα1-6(Manα1-6)Manα1-3(Manα1-3)Manβ-sp4	8.73	2.44	0.53	0.17	0.6	0.03
496	Le^b-Lac	Fucα1-2Galβ1-3(Fucα1-4)GlcNAcβ1-3Galβ1-4Glcβ-sp4	2	2.88	0.13	0.21	0.21	0.18
497	Le^y-Lac	Fucα1-2Galβ1-4(Fucα1-3)GlcNAcβ1-3Galβ1-4Glcβ-sp4	1.05	0.91	0.79	0.3	0.81	0.72
498	(LN)$_3$	(Galβ1-4GlcNAcβ1-3)$_3$-sp3	1.69	0.36	1.82	1.36	2.44	0.49
499	I	Galβ1-4GlcNAcβ1-6(Galβ1-4GlcNAcβ1-3)Galβ1-4GlcNAc-sp2	1.05	0.63	1.57	1.14	1.97	1.17
501	Gb5	Galβ1-3GalNAcβ1-3Galα1-4Galβ1-4Glcβ-sp4	4.2	2.57	2.06	1.71	2.95	0.11
502	maltohexaose	(Glcα1-6)$_6$β-sp4	5.78	2.3	0.64	0.2	0.71	0.11
503	chitohexaose	(GlcNAcβ1-4)$_6$β-sp4	1.31	1.61	0.42	0.08	0.37	0.06
504	9-OS	(A-GN-M)$_2$-3,6-M-GN-GNβ-sp4	1.36	1.98	1.2	1.01	1.55	1.14
505	7-OS	(GN-M)$_2$-3,6-M-GN-GNβ-sp4	0.53	0.52	2.32	1.7	3.38	1.24
527	3`SLN-LacNAc	Neu5Acα2-3Galβ1-4GlcNAcβ1-3Galβ1-4GlcNAcβ-sp2	3.56	5.54	0.33	0.02	0.33	0.1
528	SiaLe^x-3Gal	Neu5Acα2-3 Galβ1-4(Fucα1-	1.13	1.73	2.38	1.34	0.14	0.1

		3)GlcNAcβ1-3Galβ-sp3						
529	LSTb	Neu5Acα2-6(Galβ1-3)GlcNAcβ1-3Galβ1-4Glcβ-sp4	1.01	0.16	1.66	1.34	2.18	1.05
531	GD2	Neu5Acα2-8Neu5Acα2-3(GalNAcβ1-4)Galβ1-4Glc-sp2	1.82	0.57	1.43	0.96	1.84	0.51
532	GT3	Neu5Acα2-8Neu5Acα2-8Neu5Acα2-3Galβ1-4Glc-sp2	2.97	1.76	2.21	1.6	2.94	0.51
533	GT2	(Neu5Acα2-8)$_2$Neu5Acα2-3(GalNAcβ1-4)Galβ1-4Glc-sp2	2.28	1.28	1.06	0.02	0.98	0.33
534	6`SLN-LacNAc	Neu5Acα2-3Galβ1-4GlcNAcβ1-3Galβ1-4GlcNAcβ-sp3	0.6	0.37	0.46	0.2	0.49	0.4
536	LSTa	Neu5Acα2-3Galβ1-3GlcNAcβ1-3Galβ1-4Glcβ-sp4	0.45	0.13	1.05	0.17	1.04	0.48
537	LSTd	Neu5Acα2-3Galβ1-4GlcNAcβ1-3Galβ1-4Glcβ-sp4	2.29	0.75	0.67	0.08	0.62	0.46
538	MFLNH III	Le^{x}1-6'(Le^{c}1-3')Lac-sp4	4.59	1.19	0.61	0.19	0.45	0.16
539	MFLNH I	LacNAc1-6'(Le^{d}1-3')Lac-sp4	3.05	1.92	1.77	1.36	2.4	0.43
540	MSMFLNnH	Le^{x}1-6'(6'SLN1-3')Lac-sp4	3.41	1.41	1.51	1.19	2.07	0.38
541	DFLNH (a)	Le^{x}1-6'(Le^{d}1-3')Lac-sp4	4.63	2.01	0.21	0.22	0.31	0.04
542	MF(1-3)iLNO	Le^{c}Le^{x}1-6'(Le^{c}1-3')Lac-sp4	6.14	4.24	1.22	0.28	1.33	0.04
543	TFLNH	Le^{x}1-6'(Le^{b}1-3')Lac-sp4	3.19	2.39	0.32	0.01	0.32	0.07
625	hyaluroninc acid	(GlcAβ1-4GlcNAcβ1-3)$_8$-NH$_2$-ol	2.46	3.62	0.37	0.11	0.26	0.21
627	11-OS, YDS	(Sia2-6A-GN-M)$_2$-3,6-M-GN-GNβ-sp4	2.4	3.25	0.3	0.27	0.14	0.01
