# Supplementary material for: Blood Group Antigen Recognition via the Group A Streptococcal M Protein Mediates Host Colonization
Source: mBio. 2017 Jan 24;8(1):e02237-16. doi: 10.1128/mBio.02237-16 (PMC5263248; doi:10.1128/mBio.02237-16)
Supplement: FIG. S1 [file mbo002173156sf1.pdf]

Supplementary Figure 1

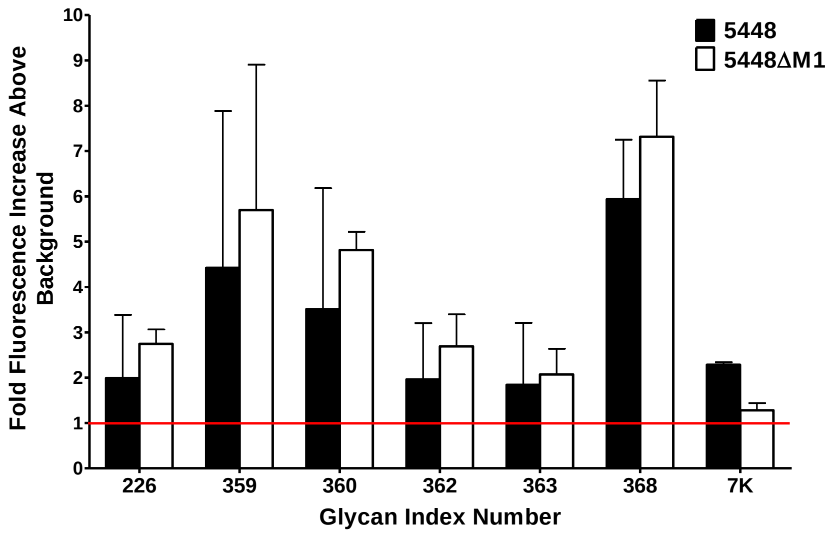

Supplementary Figure 1: Glycan binding profile of M1T1 clone GAS strains 5448 and 5448ΔM1 to blood group antigen A and B related structures. Glycan binding was analysed using ProScanArray imaging software, ScanArray Express (PerkinElmer, USA) and the data exported to Microsoft Excel for further analysis. Bacterial binding to a glycan was defined as a value $\geq$ 1 fold increase above mean background RFU. The mean background was calculated from the average RFU of all empty spots on the array plus three standard deviations. Statistical analysis of the data was performed by a Student's *t*-test with a confidence level of 99.99% ($p \leq 0.0001$) and only glycans that met these criteria for three biologically independent samples (n = 12 glycans spot replicates) were interpreted as positive binding interactions. Glycan index: 226, Galα1-3(Fucα1-2)Galβ; 359, Galα1-3(Fucα1-2)Galβ1-3GlcNAcβ; 360, Galα1-3(Fucα1-2)Galβ1-4GlcNAcβ; 362, Galα1-3(Fucα1-2)Galβ1-3GalNAcα; 363, Galα1-3(Fucα1-2)Galβ1-3GalNAcβ; 368, GalNAcα1-3(Fucα1-2)Galβ1-4GlcNAcβ; 7K, GalNAcα1-3(Fucα1-2)Gal.
