# Supplementary material for: Blood Group Antigen Recognition via the Group A Streptococcal M Protein Mediates Host Colonization
Source: mBio. 2017 Jan 24;8(1):e02237-16. doi: 10.1128/mBio.02237-16 (PMC5263248; doi:10.1128/mBio.02237-16)
Supplement: FIG. S2 [file mbo002173156sf2.pdf]

Supplementary Figure 2

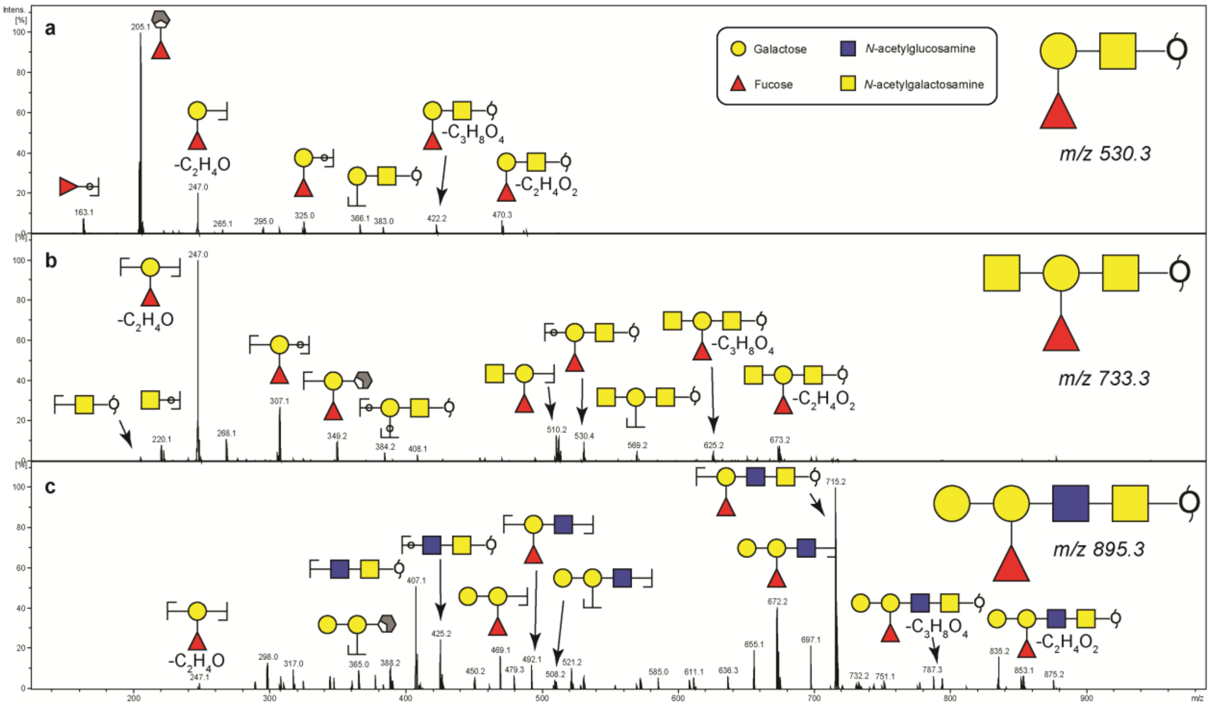

Supplementary Figure 2: Representative mass spectra of *O*-glycans released from salivary glycoproteins proteins. Collision-induced dissociation–MS/MS fragment spectra of singly charged negative ion of three *O*-linked glycan structures from saliva at (a) *m/z* 530.3^{1-}, (b) *m/z* 733.3^{1-} and (c) *m/z* 895.3^{1-}. The fragmentation spectra of these unique glycan species represented here were used to identify secretor and ABO(H) blood group status. For example, the structure with *m/z* 530.3^{1-} is indicative of an H-antigen and was observed to be present only in secretor individuals. Similarly, the fragmentation spectra of *m/z* 733.3^{1-} show an *O*-glycan structure with blood group A epitope and *m/z* 895.3^{1-} with blood group B epitope.
