# Supplementary material for: Blood Group Antigen Recognition via the Group A Streptococcal M Protein Mediates Host Colonization
Source: mBio. 2017 Jan 24;8(1):e02237-16. doi: 10.1128/mBio.02237-16 (PMC5263248; doi:10.1128/mBio.02237-16)
Supplement: FIG. S3 [file mbo002173156sf3.pdf]

Supplementary Figure 3

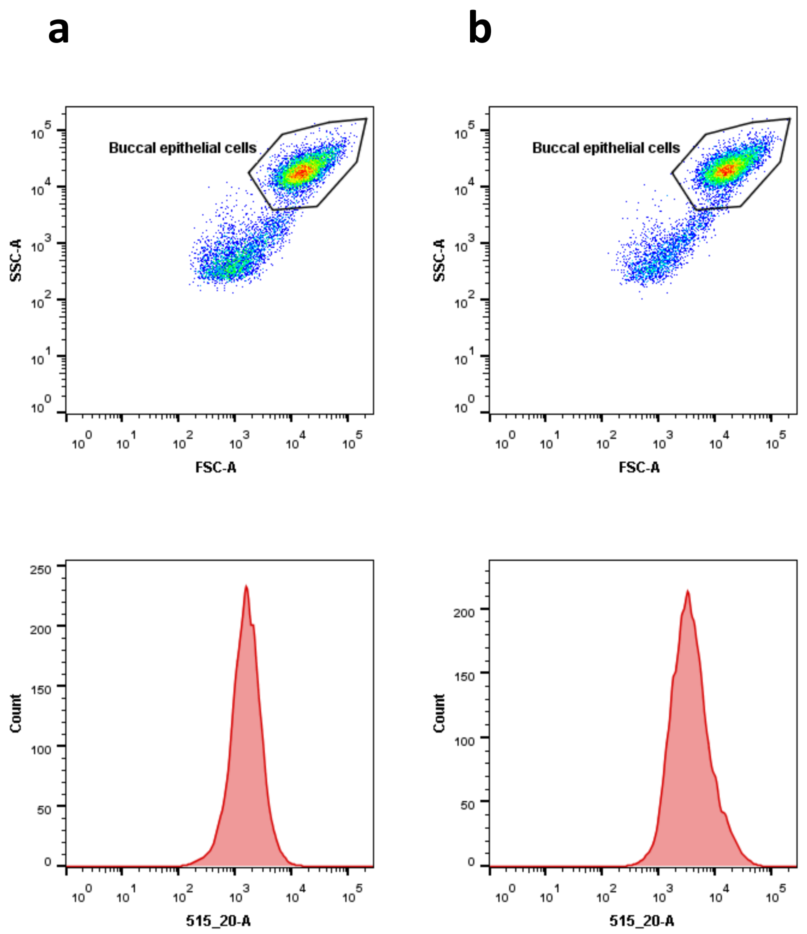

Supplementary Figure 3: Flow cytometry assay of 5448 WT binding to human buccal epithelial cells. Dot plots at the top of each panel show the forward scatter (FSC-A) and the side scatter (SSC-A) distribution while the corresponding histograms of buccal epithelial cell count vs. fluorescence intensity at 515-520 nm is shown below. (a) Auto-fluorescence of buccal epithelial cells alone. (b) 5448eGFP WT incubated with buccal epithelial cells.
