# Supplementary material for: Blood Group Antigen Recognition via the Group A Streptococcal M Protein Mediates Host Colonization
Source: mBio. 2017 Jan 24;8(1):e02237-16. doi: 10.1128/mBio.02237-16 (PMC5263248; doi:10.1128/mBio.02237-16)
Supplement: FIG. S4 [file mbo002173156sf4.pdf]

## Supplementary Figure 4

**a**

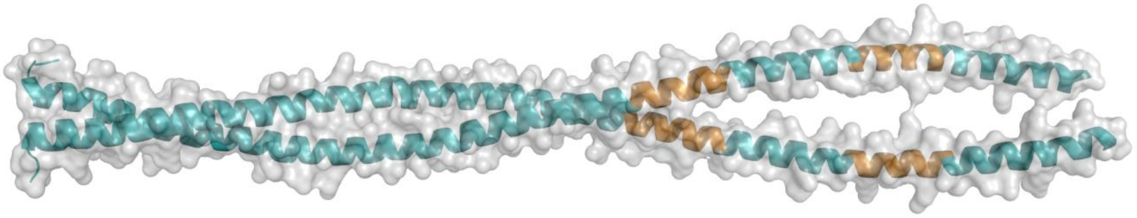

**b**

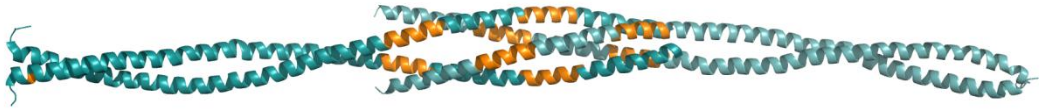

**Supplementary Figure 4.** (a) Crystal structure of the dimeric AB fragment of M1 (Protein Data Bank ID 2OTO [20]). (b) Antiparallel coiled-coil interactions between two M1AB dimers (dark and light teal) in the crystal.  The minimal motif shown to bind LNT and H-antigen type 1 is highlighted in orange. The structures are shown in cartoon representation as well as surface representation in (a). The figure was prepared using PyMol (Schrödinger, LLC).
